# Improved Photocatalyzed Degradation of Phenol, as a Model Pollutant, over Metal-Impregnated Nanosized TiO_2_

**DOI:** 10.3390/nano10050996

**Published:** 2020-05-22

**Authors:** S. Belekbir, M. El Azzouzi, A. El Hamidi, L. Rodríguez-Lorenzo, J. Arturo Santaballa, M. Canle

**Affiliations:** 1 Laboratoire de Physico-Chimie des Matériaux et Nanomateriaux, Faculté des Sciences, Université Mohammed V, Avenue Ibn Battouta, Rabat BP 1014, Morocco; belekbirsoukayna@gmail.com (S.B.); elazzouzim@hotmail.com (M.E.A.); adnane_el@gmail.com (A.E.H.); 2 International Iberian Nanotechnology Laboratory, 4715-330 Braga, Portugal; laura.rodriguez-lorenzo@inl.int; 3 Grupo React!, Departamento de Química, Facultade de Ciencias & CICA, Universidade da Coruña, E-15071 A Coruña, Spain; arturo.santaballa@udc.es

**Keywords:** phenol, photocatalysis, titania, surface impregnation, photodegradation, reaction mechanism, adsorption analysis, energy efficiency

## Abstract

Photocatalyzed degradation of phenol in aqueous solution over surface impregnated TiO_2_ (M = Cu, Cr, V) under UV-Vis (366 nm) and UV (254 nm) irradiation is described. Nanosized photocatalyts were prepared from TiO_2_-P25 by wet impregnation, and characterized by X-ray diffraction, X-ray fluorescence, transmission electron microscopy, UV-Vis diffuse reflectance spectroscopy, Raman spectroscopy, and adsorption studies. No oxide phases of the metal dopants were found, although their presence in the TiO_2_-P25 lattice induces tensile strain in Cu-impregnated TiO_2_-P25, whereas compressive strain in Cr- and V-impregnated TiO_2_-P25. Experimental evidences support chemical and mechanical stability of the photocatalysts. Type IV N_2_ adsorption–desorption isotherms, with a small H3 loop near the maximum relative pressure were observed. Metal surface impregnated photocatalysts are mesoporous with a similar surface roughness, and a narrow pore distribution around ca. 25 Å. They were chemically stable, showing no metal lixiviation. Their photocatalytic activity was followed by UV-Vis spectroscopy and HPLC–UV. A first order kinetic model appropriately fitted the experimental data. The fastest phenol degradation was obtained with M (0.1%)/TiO_2_-P25, the reactivity order being Cu > V >> Cr > TiO_2_-P25 under 366 nm irradiation, while TiO_2_-P25 > Cu > V > Cr, when using 254 nm radiation. TOC removal under 366 nm irradiation for 300 min showed almost quantitative mineralization for all tested materials, while 254 nm irradiation for 60 min led to maximal TOC removal (*ca*. 30%). Photoproducts and intermediate photoproducts were identified by HPLC–MS, and appropriate reaction pathways are proposed. The energy efficiency of the process was analysed, showing UV lamps are superior to UVA lamps, and that the efficiency of the surface impregnated catalyst varies in the order Cu > V > Cr.

## 1. Introduction

Industry development imply heavy economical charges associated to waste removal, often a cocktail of pollutants harmful to the environment, dangerous for human health, and difficult to degrade by natural mean [1]. Among common persistent pollutants, phenol derivatives are a ubiquitous group. Common sources of phenols pollution in water bodies are paints, pesticides, coal, polymers, food, cosmetic and pharmaceutical industries, resins, oil and petrochemical products. Phenol is cytotoxic, damaging the skin and mucous membranes when concentrated, while causing erythema, vesicles and ulcerations, when dilute. It may also cause peripheral neuritis, renal malfunction and liver/kidney necrosis. Moreover, phenol acts as a cardiovascular depressant. General intoxication by phenol may be severe, including possible vascular collapse, respiratory failure and death. Thus, EU directive 80/778/CE has limited phenol concentration in drinking water to 0.5 μg/L [2]. Phenol has been widely accepted as a model persistent organic pollutant in different pollution abatement studies, including photodegradation technologies.

Different methods have been used to achieve phenol safety level, in the range 0.1–1.0 mg·L^−1^ [3]: adsorption, electrochemical oxidation, biological treatment, etc. However, these processes generate byproducts that may be harmful, making additional treatments necessary, implying further costs [4]. Additionally, these methods cannot give satisfactory yields in terms of pollutant removal because of the solubility of phenol in water. Therefore, it is essential to develop modern technologies for efficient and cheaper treatments [5]. Photocatalysis is one of the most promising methods for complete mineralization of persistent organic pollutants like phenol and derivatives, thus avoiding generation of secondary pollutants [1,4]. In brief, photocatalytic degradation of pollutants involves formation of e^−^/h^+^ pairs upon irradiation of the semiconductor (SC) with photons of energy higher than or equal to the band gap energy (hυ ≥ Eg). An electron is excited from the valence band (VB) to the conduction band (CB) of the SC, yielding an oxidation site (hole, h^+^) and a reduction site (electron, e^−^). Holes, h^+^, may oxidize adsorbed organic species, water or HO^–^, forming strongly oxidant hydroxyl radicals, HO^•^, or organic radical cations, R^•+^. Electrons, e^−^, may reduce O_2_ to the superoxide radical anion, O_2_^–^, or organic species to the corresponding radical anions, R^–^. Finally, degradation of organic compounds C_x_H_y_O_2_ into CO_2_ and H_2_O takes place after reaction with HO^•^, O_2_^•–^, or breakage of R^+^ or R^–^ [6].

A number of SCs have been tested in heterogeneous photocatalysis: TiO_2_, ZnO, ZnS, WO_3_, GaP, Fe_2_O_3_, CdS, etc. [7,8]. The most extensively used photocatalyst is TiO_2_ which presents many advantages compared to others: it is abundant, inexpensive, stable, efficient and non-toxic [9]. The most effective form of TiO_2_ for heterogeneous photocatalysis is the commercial titania mixture Evonik TiO_2_-P25. Its very high photocatalytic activity is due to the anatase-rutile junction that reduces the rate of e^−^/h^+^ recombination [10]. Despite its excellent photocatalytic performance, it shows a number of drawbacks: (i) it requires excitation wavelengths shorter than 415 nm, as the overlap between sunlight emission and TiO_2_-P25 absorbance is very low, ca. 4% UV and (ii) e^−^/h^+^ recombination is large, limiting its photoactivity. Other SCs (e.g., CdS or GaP) have advantages such as absorbing larger fraction of sunlight as compared to TiO_2_, however, they undergo photocorrosion during the photocatalytsis. Different strategies have been developed to improve Vis light absorption and/or to reduce e^−^/h^+^ recombination. Among these, doping and impregnation with transition metal ions lead to an improvement in photocatalytic activity [11,12] through the generation of intermediate energy states in the band gap of TiO_2_ (increasing Vis light absorption) or trapping of photoexcited electrons (reducing e^−^/h^+^ recombination) [13]. Photocatalysts have been used for pollution abatement in water, both in suspension and immobilized over suitable supports. Alternative strategies, such as doping TiO_2_ onto large particles avoid the expensive cost of nanofiltration in real-world environmental applications [14]. Here, we focus on the behavior of suspended photocatalysts, leaving its immobilization for a later stage.

In this study, we have impregnated TiO_2_-P25 with different amounts of metals (Cu, Cr, and V), to improve visible light harvesting, and investigated the variables controlling phenol photodegradation, as a model of phenolic pollutants abatement, by heterogeneous photocatalysis with the resulting materials under Vis and UV light. 0.1%, 0.3%, 0.5%, and 1% of Cu, Cr, and V were used, and the corresponding reaction mechanism for the phenol photocatalyzed degradation was described.

## 2. Experimental

### 2.1. Materials

TiO_2_-P25 was purchased from Evonik (*ca*. 70:30% anatase: rutile with a small amount of amorphous phase and a surface area of 55 ± 15 m^2^·g^−1^) [15]. Copper (II) sulfate pentahydrate (CuSO_4_ 5H_2_O) (≥98%, Sigma), chromium (III) nitrate (Cr(NO_3_)_3_·9H_2_O (≥98%, Sigma), ammonium metavanadate NH_4_VO_3_ (99.996%, Sigma), and phenol (C_6_H_5_OH) (99.5% Sigma-Aldrich) were purchased and used without further purification. Acetonitrile was purchased for J.T. Baker with HPLC grade. O_2_ (purity ≥ 99.995%) gas was used in some experiments. Distilled water used in the experiments was obtained from a Millipore apparatus (Milli-Q water) with a resistivity of 18.2 MΩ at 298.0 K and total organic carbon (TOC) less than 5 μg·L^−1^.

### 2.2. Catalyst Synthesis

An incipient wetness impregnation method was adopted for metal immobilization. The desired amount of metal salt was dissolved in distilled water, to which 1 g of TiO_2_-P25 was added. The mixture was then kept under vigorous stirring at 50 °C for 24 h. This suspension was dried at 50 °C. Finally, the photocatalysts were calcined at 500 °C for 4 h with a ramp rate of 100 °C/h. The resulting Cu/TiO_2_, Cr/TiO_2_, and V/TiO_2_ photocatalysts were thoroughly ground and labeled as M(X%)/TiO_2_ where M stands for the metal and X represents its mass percentage (0.1%, 0.3%, 0.5%, and 1%). To test the stability of the photocatalysts, they were suspended in distilled water for 2 h, with mechanical stirring, and the filtrate composition was analysed for the presence of the corresponding metal cation.

### 2.3. Characterization Techniques

The surface morphology of TiO_2_-P25 and the different M(X%)/TiO_2_ was recorded using Transmission Electron Microscopy (TEM). The samples were prepared by depositing drops of nanoparticle solutions on carbon formvar coated copper grids (electron microscopy, 200 mesh) and air drying. TEM images were obtained with a Jeol JEM 1100 instrument operating at an acceleration voltage of 80 kV the carbon content was studied by elemental analysis (Thermo Flash 1112). X-ray diffraction (XRD) measurements were carried out on a Bruker Siemens D5000 diffractometer with Bragg–Brentano geometry and θ/2θ configuration, equipped with a graphite monochromator. The optics consist of 2° primary and secondary Soller slits, variable output slit, 1 mm reception slit, 0.2 mm monochromator slit and 0.6 mm detector slit. The detector was a scintillation counter. The conditions of acquisition were: sweeping range (2θ): 2–80°, skip size (step size): 0.050°, acquisition time in each jump (time per step) 2.5 s. DiffracPlus v. 8.0.0.2 (Socabim) software was used for data processing.

The anatase mass fraction in the synthesized metal impregnated photocatalysts was calculated from XRD data using the Spurr and Myers Equation (1): [16]

(1) $$f_{A}=\frac{1}{1+1.265\frac{I_{R}}{I_{A}}}$$

where *f*_A_ is the mass fraction of crystalline anatase in the TiO_2_-P25 nanoparticles, *I*_R_ and *I*_A_ are the intensity of the (110) rutile and of the (101) anatase reflection, respectively.

The Scherrer Equation (2): [17]

(2) $$\tau =\frac{K\cdot \lambda }{\beta \cdot \cos\theta }$$

was used to calculate the crystallite size (*τ*), where k is a constant (0.89), *λ* is the X-ray wavelength, *β* is the full width at half maximum (FWHM) of the diffraction line and *θ* is the diffraction angle. *θ* and *β* were taken for (1 0 1) and (1 1 0) crystal plane of anatase and rutile phase, respectively. The contribution of size and strain to peak broadening was estimated using the Williamson–Hall (W–H) Equation (3): [18]

(3) $$\beta_{hkl}\cdot \cos\theta =\frac{K\cdot \lambda }{\tau }+4\cdot \varepsilon \cdot sin\;\theta$$

where ε is the microstrain, and also with the size–strain plot (SSP) in accordance with the Equation (4): [19]

(4) $${\left(d_{hkl}\cdot \beta_{hkl}\cdot \cos\theta \right)}^{2}=\frac{K’}{\tau }\cdot \left(d_{hkl}^{2}\cdot \beta_{hkl}\cdot \cos\theta \right)+{\left(\frac{\varepsilon }{2}\right)}^{2}$$

where K’ is a particle shape dependent constant, e.g., 0.75 for spheres.

The composition of the catalyst after its use was tested by semi quantitative X-ray fluorescence on a S4 Pioneer Bruker X-ray spectrofluorometer, equipped with Rh/Ag tube and analyzer crystals LiF200, Ge, PET, OVO-8 and OVO-55. Raman measurements of the dried samples on glass were performed using a Witec Alpha 300 R confocal Raman system equipped with a 633 nm excitation laser line (10× objective), holographic 600 gr⋅mm^−1^ grating and Peltier-cooled CCD detector (–70 °C). Raman spectra were acquired at room temperature over a total spectra range of 90–2800 cm^−1^ (2.7 cm^−1^ spectrum resolution) with ten 2 s accumulations and laser power at the sample of 21 mW. The laser was focused onto the sample by using a 10× objective (N.A. 0.2) providing a laser spot of ca. 3.8 µm. The Raman band of a silicon wafer at 520 cm^−1^ was used to calibrate the spectrometer. A simple baseline (as vertical setoff; y = 0) were applied to each spectrum using Spectragryph 1.2.11. The spectra were normalized to the E_g_ peak (142 cm^−1^) for a better comparison between samples The positions and widths of the peaks were extracted by fitting the spectrum with pseudo-Voigt functions using Project FOUR (^®^ 2014, Witec GmbH, Ulm, Germany).

The UV-Vis diffuse reflectance (DRS) spectra (200–800 nm) of solid photocatalysts were measured on a JASCO V-560 UV-Vis spectrophotometer with a double monochromator and double beam optical system, equipped with an integrating sphere attachment (JASCO ISV-469, Oklahoma City, OK, USA). Reflectance spectra were converted by the instrument software (JASCO) to equivalent absorption Kubelka–Munk units. BET surface areas of the photocatalyst samples were measured using a BET equipment Tristar II Plus (Micromeritics; automatic station with 3 simultaneous measurement ports). The isotherms were measured in the range *P*/*P*_0_ = 0.1–1.0. The gases used were He (for the measurement of the dead volume of the sample holders) and N_2_ as adsorption gas. The measurements were made at the temperature of liquid nitrogen (77.4 K). The BET zone range used for the calculation of the specific surface area was *P*/*P*_0_ = 0.05–0.3. The software used for control, acquisition and data processing was “Microactive for Tristar II Plus”, v.2.03 (Micromeritics). To test the stability of the photocatalysts, the filtrate composition was analyzed by ICP-MS (Perkin-Elmer model NexION 300D).

The topography of the metal impregnated photocatalysts was calculated as roughness exponent, also known as fractal dimension D_S_, using the Frenkel–Halsey–Hill Equation (5): [20,21,22,23]

(5) $$\ln S^{lg}=const-\left(3-D_{S}\right)\cdot \ln\mu$$

where *S^lg^* is the adsorbed amount of nitrogen at the relative pressure *P*/*P*^0^, D_S_ relates to solid roughness and its adsorption and permeability capacity, and μ is the adsorption potential (Equation (6)):

(6) $$\mu =R\cdot T\;\cdot \ln\frac{P^{0}}{P}$$

### 2.4. Photocatalytic Activity

**Figure 1:**
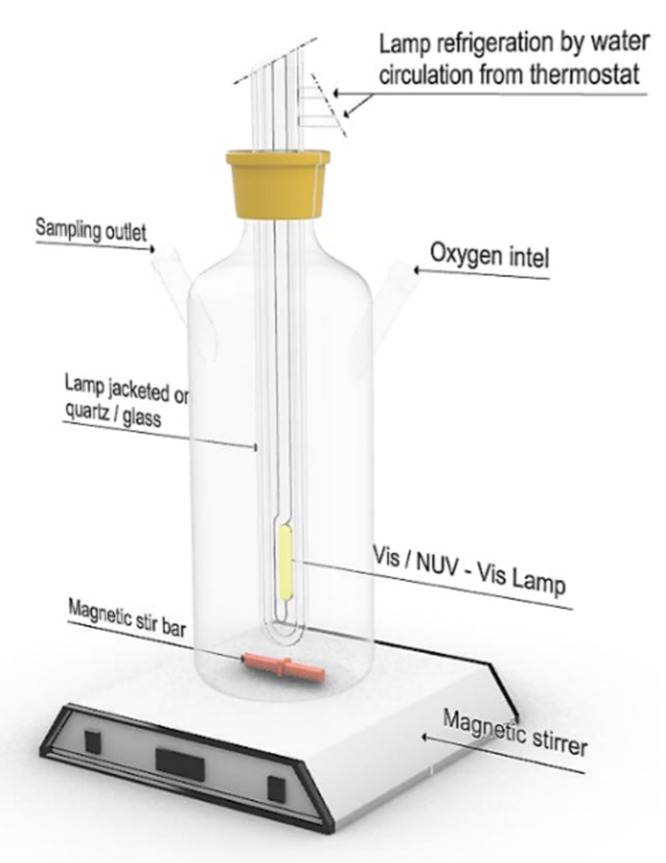
Scheme of the photoreactor used in heterogeneous photocatalysis experiments.

The photocatalytic activity under UV and near UV-Vis light (NUV-Vis) of the synthesized photocatalysts was tested by monitoring the concentration changes of an aqueous phenol solution (C_6_H_5_OH) in an annular immersion photoreactor (shown schematically in Figure 1).

Experiments under NUV-Vis irradiation were carried out with a medium-pressure Hg vapour lamp, with intense emission lines at *λ*_exc_ = 254, 313, 366, 405, 436, 546, and 578 nm. UV lines at *λ*_exc_ < 366 nm were filtered using a DURAN 50^®^ glass jacket filled with water. The photon flux at 366 nm, as determined by potassium ferrioxalate actinometry [24], was 2.38 × 10^–6^ Einstein·s^−1^. Experiments under UV irradiation were carried out with a low-pressure Hg vapor lamp, with a single intense emission line at *λ*_exc_ = 254 nm, located axially in the reactor inside a quartz tube. The photon flux at 254 nm, as determined by potassium ferrioxalate actinometry [24], was of 8.33 × 10^–8^ Einstein·s^−1^.

Unless otherwise stated, the different reactions were carried out with 200 mL of 50 ppm phenol solutions and 200 mg of photocatalyst, in all cases in the presence of O_2_, under magnetic stirring. The concentration of O_2_ was routinely tested on the water used, and it was according to the expected solubility at the experimental temperature. All photocatalyst suspensions were allowed to equilibrate in the dark for 30 min as we know from our previous work that this time is sufficient to allow the establishment of the adsorption–desorption equilibrium [25].

The different heterogeneous suspensions were irradiated for 60 min under UV light or for 300 min under NUV-Vis light. Aliquots were withdrawn at given time intervals, and filtered through Sartorius NY 0.45™ filters, for phenol and total organic carbon (TOC) analysis. All kinetic runs were performed at 298.0 K, the temperature being maintained by water flow from a thermostat–cryostat. The pH of the medium was the natural pH of the solution, given by the mixture of the photocatalyst and phenol (pH(Cu/TiO_2_) = 4.7, pH(V/TiO_2_) = 4.9, pH(Cr/TiO_2_) = 5.0).

The photocatalytic degradation efficiency was calculated based on the initial phenol concentration. [Phenol] was monitored by measuring the UV-Vis absorbance at 270 nm, using a Biochrom Libra S70 spectrophotometer, and by UV-Vis HPLC analysis at 210 and 270 nm, in a Thermo Fisher apparatus, equipped with a 6000 LP UV detector, an AS 3000 autosampler and a P4000 solvent pump. A Kromaphase C18 column (4.6 mm × 150 mm × 5 μm) was used, with an injected volume of 50 μL, a flow rate of 1.0 mL·min^−1^, at 30 °C, with acetonitrile: water (25:75, *v/v*) as mobile phase. The TOC removal efficiency was measured using a ShimadzuTOC-5000A analyzer.

Photoproducts were identified using HPLC/MS (Thermo Scientific LTQ Orbitrap Discovery apparatus), equipped with an electrospray interface operating in negative ion mode (ESI-). A Phenomenex Kinetex XB-C18 column (100 mm × 2.6 μm) was used, operated at 30 °C with elution solvents A (0.1% formic acid) and C (0.1% methanol.) at flow rate of 200 μL·min^−1^. The gradient was as follows: 0–1 min, 95–95% A and 5–5% C; 1–8 min, 95–5% A and 5–95% C; 8–10 min, 5–5% A and 95–95% C; 10–11 min, 5–95% A and 95–5% C; 11–15 min, 95–95% A and 5–5% C. Typical injection volumes were 5–25 μL. The analyses were carried out using full-scan data dependent MS scanning from m/z 50 to 500.

## 3. Results and Discussion

### 3.1. Characterization of the Catalysts

The efficiency of a photocatalyst is related to superficial and structural properties of the semiconductor such as its crystalline structure, surface area, particle size distribution, porosity, band gap, and density of surface hydroxyl moieties [26].

#### 3.1.1. TEM

**Figure 2:**
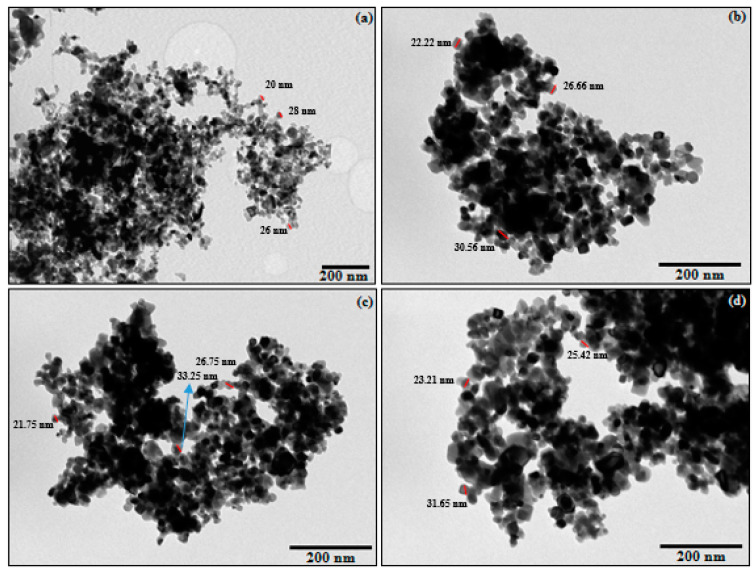
Transmission electron microscopy (TEM) micrographs of (**a**) TiO_2_ –P25, (**b**) Cu(0.1%)/TiO_2_, (**c**) V(0.1%)/TiO_2_, and (**d**) Cr(0.1%)/TiO_2_.

Surface morphologies of TiO_2_ –P25 and the different M(X%)/TiO_2_ were studied using Transmission electron microscopy (TEM). Typical results are shown in Figure 2.

The TiO_2_-P25 sample (Figure 2a) showed mainly homogeneous particles with quite similar morphologies of nanometric size, ranging from 20 to 35 nm. Similar results were observed for M(0.1%)/TiO_2_ photocatalysts (Figure 2b–d). Regular shapes were observed in all cases, with similar edges, which is compatible with a common crystalline system, corresponding to the main components of TiO_2_-P25, anatase and rutile, as shown by XRD analysis and Raman spectroscopy (see below). Though the observed crystals appear a bit larger than for the non-impregnated sample, this is not attributed to a crystallite-size effect, as will be discussed below in the XRD section (see below).

Instead, formation of large agglomerates, with sizes between 200 and 600 nm, was observed in all cases, with a higher incidence for Cu(0.1%)/TiO_2_ as demonstrated qualitatively by scanning electron microscopy (SEM) images at low magnification (Figure S1 of Supplementary Information). The homogeneity of the system and the nanometric dimensions play an important role in the photoactivity of a semiconductor catalyst since it influences the electron/hole recombination process [27].

Since the three impregnated photocatalysts do not show relevant morphological changes relative to TiO_2_-P25, similar photocatalytic behavior could be anticipated for them.

#### 3.1.2. X-ray Diffraction

**Figure 3:**
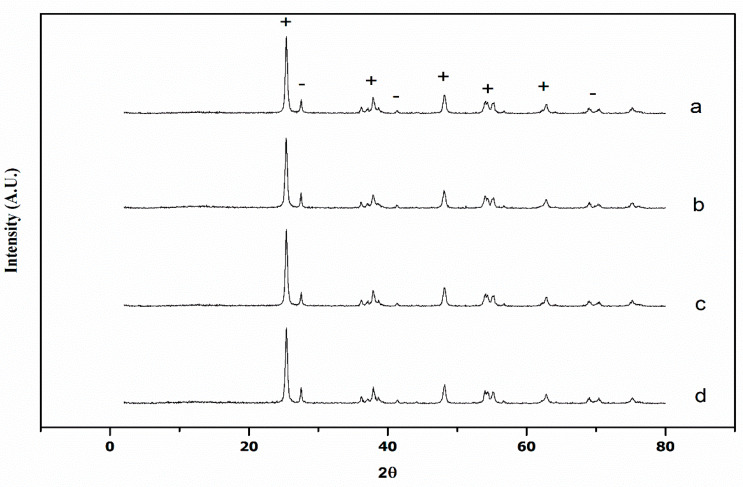
X-ray diffraction patterns of TiO_2_ and various samples containing 0.1% of transition metal: (**a**) TiO_2_-P25, (**b**) Cu/TiO_2_, (**c**) V/TiO_2_, and (**d**) Cr/TiO_2_. +: anatase, −: rutile.

The observed X-ray diffraction pattern of TiO_2_ and M(X%)/TiO_2_ samples is shown in Figure 3.

The observed diffraction peaks were assigned to both anatase TiO_2_ (JCPDS 89-4921), marked ‘+’, and rutile TiO_2_ (JCPDS 65-191) marked ‘-’. The corresponding diffraction planes are shown in Table SI01 of the Supporting Information. The diffraction patterns of Cu(0.1%)/TiO_2_-P25, Cr(0.1%)/TiO_2_-P25 and V(0.1%)/TiO_2_-P25 catalysts were very similar to TiO_2_-P25. These results are typical to the bicrystalline structure of TiO_2_-P25, which is composed of ca. 80% anatase and 20% rutile [28], and the rutilization faintly increases in the order V > Cr > Cu (Table 1). The slight shift of diffraction angle suggests a slight lattice distortion relative to non-impregnated TiO_2_-P25 peaks (Table S1 at the Supporting Information).

Only diffraction peaks of the anatase and rutile phases have been found, none belonging to metal oxides, therefore impregnated metals are uniformly distributed on the TiO_2_-P25 surface. It has been reported that only above 65.97 wt.% Cu in TiO_2_ significant cooper oxide peaks can be observed in XRD diffraction patterns [29], whereas full surface coverage is obtained at >5 at.% Cu [30]. V incorporates to the lattice at low V/Ti ratio and at higher loadings as V_2_O_5_ [31,32]. As observed here, no Cr phases have been reported for Cr(0.001–1%)/TiO_2_(rutile) [33].

Crystallite size values using the Scherrer Equation [17] are listed in Table 1, they are in perfect agreement with TEM observations (see above). Scherrer’s crystallite size of the anatase phase is similar for the three photocatalysts (*ca.* 22 nm), M(1%)/TiO_2_-P25. Those values are similar to that reported in the literature, e.g., V(0.1%)-TiO_2_ (anatase) [34,35] 28.4 nm. Larger sizes occur for the rutile phase, the order being V(0.1%)/TiO_2_-P25 > Cu(0.1%)/TiO_2_-P25 > Cr(0.1%)/TiO_2_-P25.

XRD peaks broadening is not only due to particle size, strain also plays a role. The simplest model to take size and strain effects into account is the Uniform Deformation Model (UDM), based on the Williamson–Hall equation (see Section 2.3) [18], which assumes that crystals are isotropic. Crystallite sizes obtained using the W–H method (see Table 1) follow the same general trend observed for the Scherrer ones, and are in the same range, again with larger values for the rutile phase.

The very small slopes of the W–H equation suggest a high degree of crystallinity, having opposite sign for Cu and for Cr- and V-impregnated photocatalysts. From there the corresponding strain (ε) values have been calculated using the W–H equation, see Section 2.3. The positive ε values, Table 1, obtained for both phases of Cu(0.1%)/TiO_2_-P25 indicate the presence of tensile strain [36] in this photocatalyst, and, on the contrary, compressive strain occurs in both phases of V(0.1%)/TiO_2_-P25 and Cr(0.1%)/TiO_2_-P25, likely reflecting the effect of the metals ionic radii on the TiO_2_ lattice. Cr^3+^ and V^5+^ ions substitute Ti^4+^ sites as those ions have similar radii (Ti^4+^ ≈ Cr^3+^ > V^5+^), whereas Cu^2+^ ions might locate in interstitial positions of the lattice due to its higher size, 0.87 Å vs. 0.745 Å of Ti^4+^. Crystallite size and strain were calculated using an average SSP method, see Section 2.3. Size, although obviously smaller than those calculated using Scherrer and W–H, shows similar trend to that of W–H equation, i.e., larger values obtained for rutile phase. Size differences relative to Scherrer and W–H model probably due to the fact that those photocatalysts are non isotropic; here crystallite size was calculated assuming isotropic and spherical crystals, i.e., K’ = ¾ in the size–strain equation. Tensile strain was found for both phases of Cu(0.1%)/TiO_2_-P25, and compressive strain for both phases of V- and Cr-impregnated photocatalysts, but for anatase phase of Cr(0.1%)/TiO_2_-P25. This result should be taken with precaution, as the isotropy of the crystals is not confirmed.

The XRD of the photocatalysts were also recorded after 2 h of stirring of photocatalyst suspension in distilled water to check its stability. The obtained XRD patterns are similar to that of TiO_2_-P25, see Table SI01 in the Supporting Information. Minor or no changes have been found in the position of the diffraction peaks, the d-space, the crystallite size and the anatase mass fraction after stirring the photocatalysts suspension in water for two hours (SI01 at the Supporting Information). The filtrate was analyzed by ICP/MS, and the results showed the stability of the photocatalysts, with only traces or ultratraces of the impregnated metals lixiviated after 2 h stirring: ca. 1% for V and Cr, and <0.02% for Cu. X-ray fluorescence (XRF) results are in line with this, the semiquantitative analysis of the surface did not show any variation after 2 h of stirring of the photocatalyst in water. A second and third periods of 2 h of stirring led only to ultratraces being detected, below the quantification limit of the technique. Similar results were obtained when the photocatalysts were used for photodegradation of phenol in three repeated cycles.

#### 3.1.3. Raman Spectroscopy

**Figure 4:**
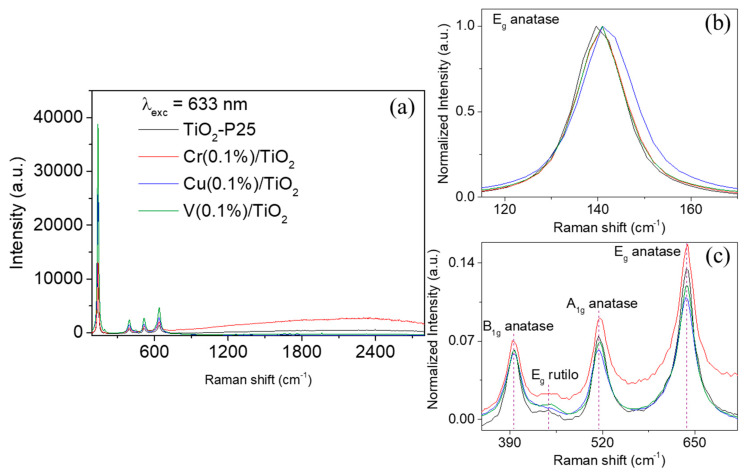
Raman spectra of air-dried non-impregnated TiO_2_ (black spectrum), Cr(0.1%)/TiO_2_ (red spectrum), Cu(0.1%)/TiO_2_ (blue spectrum), and V(0.1%)/TiO_2_ (green spectrum) sample on glass upon excitation at 633 nm laser line. (**a**) Full Raman spectra; (**b**) expanded spectral window from 110 to 170 cm^−1^, which a slight red-shift is observed on E_g_ Raman mode at impregnated samples. This red-shift is slightly clearer on Cu(0.1%)/TiO_2_ (blue spectrum). (**c**) Spectral window from 350 to 710 cm^−1^, showing that A_1g_ peak of anatase is slightly shifted and broadened in impregnated samples. Raman spectra shown in (**b**) and (**c**) were normalized at maximum intensity of E_g_ Raman band for the sake of comparison.

Figure 4 shows the Raman spectra of TiO_2_ and M(0.1%)/TiO_2_ samples. The anatase phase was clearly identified in all the Raman spectra. This phase shows a tetragonal structure with six active Raman modes: E_g_ (144, 197, and 639 cm^−1^), B_1g_ (399 and 519 cm^−1^), and A_1g_ (519 cm^−1^).

In these spectra, a very low-intensity band centred approximately at 455 cm^−1^ was also observed, which is related with the E_g_ mode of the rutile phase, a tetrahedral crystal structure with four active Raman modes: B_1g_ (144 cm^−1^), E_g_ (448 cm^−1^), A_1g_ (612 cm^−1^), and B_2g_ (827 cm^−1^) [37]. These five Raman peaks are characteristic of TiO_2_-P25, as a mixture of anatase (80%) and rutile (20%) [38]. In the case of M(0.1%)/TiO_2_ spectra, no extra Raman peak that could be assigned to corresponded metal oxide was observed (i.e., Cr_2_O_3_ at 296 (E_g_), 350 (E_g_), 528 (E_g_), 554 (A_1g_), and 615 (Eg) [39] CuO at 297 (A_g_), 344 (B_g_), and 629 cm^−1^ (B_g_) [40], and V_2_O_5_ typically at 285 (B_2g_), 703 (B_2g_), and 997 cm^−1^(A_g_) [41], in agreement with the XRD results (see above sub-Section 3.1.2). This feature indicates that the impregnated metal does not exist as a separate crystalline oxide phase [42].

Figure 4a shows an increase of the Raman peaks as a function of the nature of the metal cation: V >> Cu >> Cr ≈ non-impregnated, which can be attributed to an enhancement of the crystallinity (i.e., total symmetry of the TiO_2_ molecular structure) of the anatase phase by impregnation mainly with Cu and V cations [43]. The spectrum of Cr(0.1%)/TiO_2_ also showed larger luminescence background than non-impregnated TiO_2_ sample. The luminescence background disappeared in the case of Cu(0.1%)/TiO_2_ and V(0.1%)/TiO_2_. This optical relaxation, i.e., luminescence, is due to defects in the crystals [44]. Therefore, larger luminescence background in Cr-impregnated sample is due to increased structural distortions of the TiO_2_ crystal in the presence of Cr^3+^, which induce weak optical absorption of the 633 nm laser excitation during the Raman measurements.

Furthermore, a slight red-shift was observed on the E_g_ (140 cm^−1^) peak (Δ_Raman-shift_ = 2.8–4.4 cm^−1^) in M(0.1%)/TiO_2_ samples, being more evident in the case of Cu(0.1%)/TiO_2_ (Figure 4b). In the B_1g_ (512 cm^−1^) peak was also red-shifted (Δ_Raman-shift_ = 1.4–3.6 cm^−1^), but in this case the most important was observed for Cr(0.1%)/TiO_2_ (Figure 4c). It is well-known that substitution of Ti^4+^ by a dopant with lower oxidation state, Cu^2+^ or Cr^3+^ here, causes the generation of oxygen vacancies to conserve local change neutrality within the anatase-lattice. As a consequence of this structural distortion, Raman active peaks of anatase are shifted and broadened [40].

#### 3.1.4. UV-Vis Diffuse Reflectance Spectroscopy

**Figure 5:**
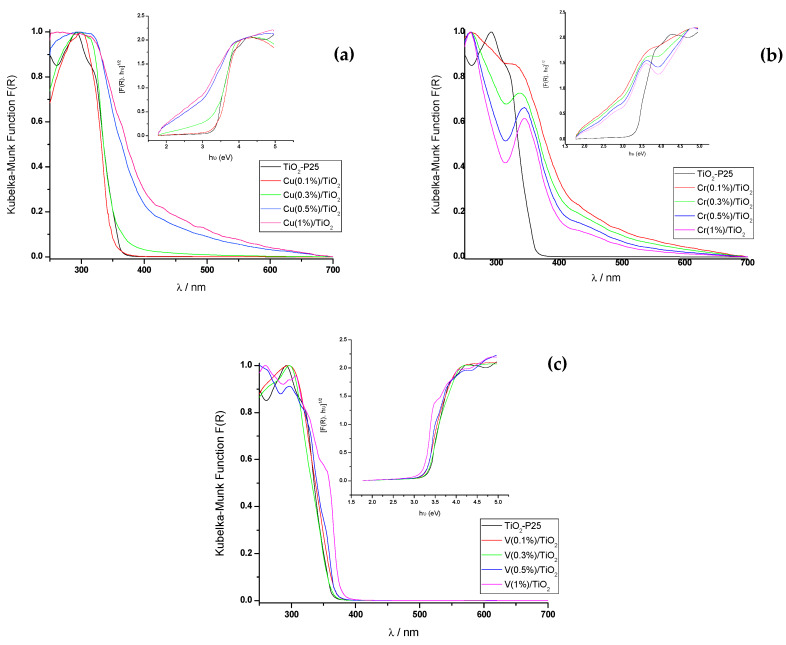
UV-Vis diffuse reflectance (DRS) spectra of M(X%)/TiO_2_ photocatalysts. M: (**a**) Cu, (**b**) Cr, and (**c**) V.

The UV-Vis DRS spectra of Cu–TiO_2_, Cr–TiO_2_, and V–TiO_2_ photocatalysts are displayed in Figure 5. TiO_2_-P25 shows an absorption peak at ca. 300 nm. The absorption band of TiO_2_-P25 from 200 to 400 nm is ascribed to O^2–^ (2p) → Ti^4+^ (3d) transitions in the tetrahedral symmetry [45]. A red shift of the absorption edge is observed for all metal surface-impregnated photocatalysts, although less pronounced for V–TiO_2_. The electronic configuration, the energy level, the concentration of the dopants and the applied light intensity play a role in the red shift of the absorption edge into the visible region [46]. Localized states within the band gap and/or oxygen vacancies and radicals associated with the impregnated metals in the TiO_2_-P25 lattice are responsible of the red shift. The different valence states of metal ion dopants relative to Ti^4+^ is consistent with the generation of oxygen vacancies, giving rise to color centers [47].

Linear extrapolation in the corresponding Tauc plots allows to obtain the band gap (E_g_) for the different photocatalysts. The corresponding optical bands were typical of semiconductors with an indirect band gap of the allowed transition type (r = 2 for the exponent of the ordinate (F(R)·h·ν)^1/r^ of the insets in Figure 5, and no light emission was observed) [48,49].

The estimated band gap for P25 TiO_2_ was 3.3 eV, in agreement with the value reported in the literature [50,51]. The obtained values are summarized in Table 2, which also show other reported values, sometimes not coincident as they heavily depend on the synthetic method.

The extended absorption of Cu-impregnated TiO_2_-P25 towards the visible region, between 350 and 550 nm, increases with Cu concentration, such behavior also described in the literature [30], can be assigned to Cu^2+^ and Cu^+^ oxidation states [51], and attributed to a charge transfer transition from O 2p to d-states of Cu oxide species, mainly as superficial amorphous CuO-like structure [52,53,54]. The appearance of this band affected the value of the band gap that decreased drastically to 2.4 and 2.0 eV for Cu(0.5%)/TiO_2_-P25 and Cu(1%)/TiO_2_-P25, respectively (Table 2), which is consistent with previous literature reports [55]. Such band gap reduction is due to the d orbitals of Cu, under the TiO_2_ CB that are able to receive electrons from the TiO_2_ VB [56].

Theoretical calculations suggest the band gap narrowing with Cu content increase due to structure distortions and formation of oxygen vacancies when Cu^2+^ ions substitute Ti^4+^ ions. New electronic states in the VB resulting from the covalent interaction between Cu and O [40].

The red shift of the absorption edge is also observed to increase with Cr content in Cr-impregnated TiO_2_-P25 photocatalysts, where both Cr^3+^ and Cr^4+^ exist [23], the latter related with heating up to 500°C (see Section 2.2). The band observed around 350 nm becomes more pronounced and shifts to higher wavelength as Cr^3+^ content increases. It is attributed to ^4^A_2g_→^4^T_1g_ of Cr^3+^ in an octahedral environment; whereas the broad absorption band around 400–700 nm can be assigned to Cr^3+ 4^A_2g_→^4^T_2g_ d–d transitions [33,57]. Cr/TiO_2_-P25 photocatalysts displayed lower band gaps compared to pure TiO_2_-P25 sample [58], the band gap decreases one eV in going to TiO_2_-P25 to Cr(0.5%)/TiO_2_, and increases 0.13 eV at Cr(1%)/TiO_2_ (Table 2).

The small red shift of the absorption edge observed for V/TiO_2_-P25 also increases with V content [31], it is the result of the electron transition from the VB (O 2p) to the t_2g_ level of V 3d orbital, located at the bottom of the TiO_2_ CB [32]. No noticeable spectral changes were observed in V/TiO_2_-P25 samples. Hence, the band gap energy was almost constant after impregnation with V ions (Table 2).

Theoretical calculations suggest band gap reduction in in V and Cr-impregnated P25 is due to the existence of V and Cr 3d orbitals between the VB and CB of Cr-doped P25, although in the case of V the 3d orbitals are adjacent the conduction band minimum (CBM) so the reduction of E_g_ relative to non-impregnated TiO_2_-P25 is lower [59].

#### 3.1.5. Textural Properties

**Figure 6:**
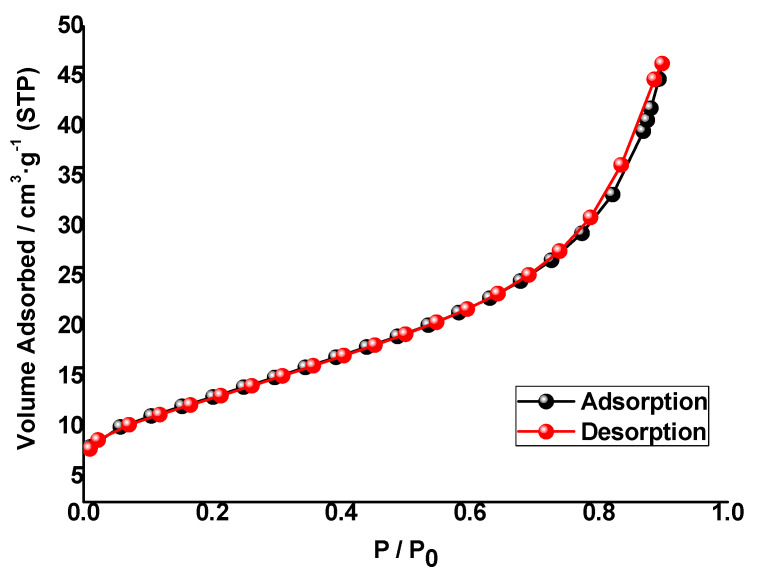
N_2_ adsorption–desorption isotherm of Cu(0.1%)/TiO_2_ photocatalyst.

**Figure 7:**
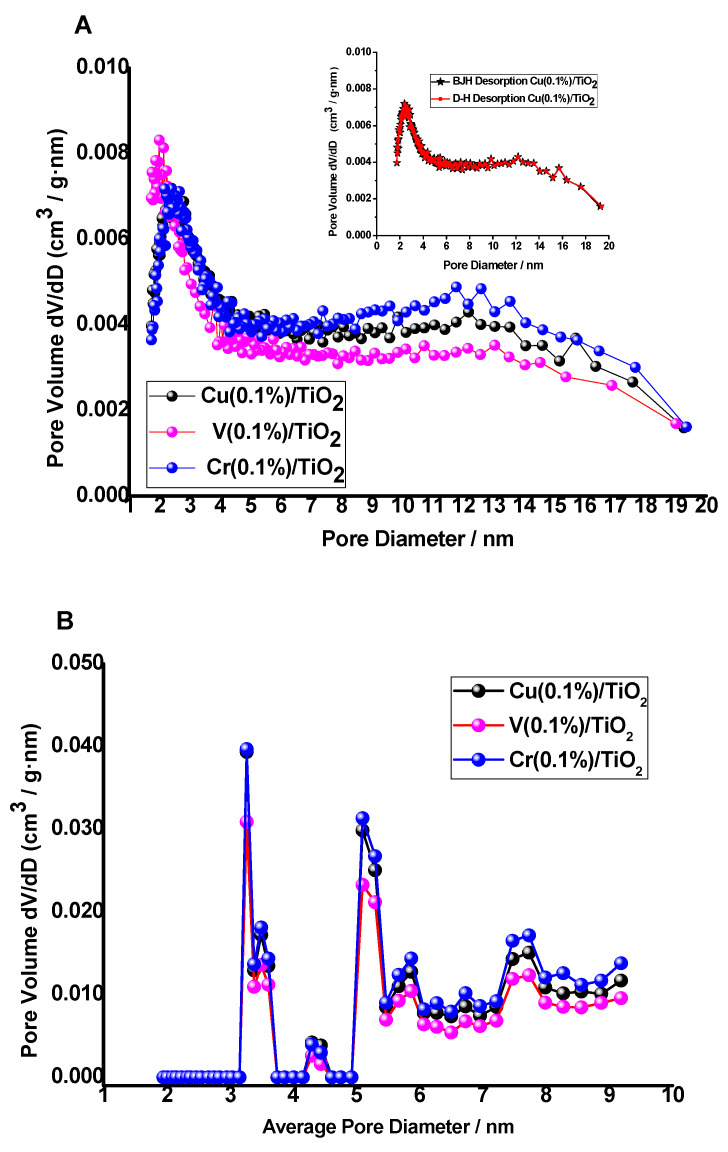
Differential specific pore volume vs. pore width distribution. (**A**) Barrett, Joyner, and Halenda (BJH) model using desorption branch. Inset: Comparison using BJH and D–H models (desorption). (**B**) 2D-non-local density functional theory method (NLDFT) model (N_2_-Carbon Finite Pores, Aspect Ratio 6, Standard Slit).

N_2_ adsorption–desorption isotherms of V(1%)/TiO_2_, Cu(1%)/TiO_2_, and Cr(1%)/TiO_2_ photocatalysts (Figure 6, Figures S8 and S9) belong to type IV, according to the IUPAC classification [60], with a small H3 hysteresis loop, which suggests these photocatalysts are mesoporous. H3 hysteresis loops are typical of mesoporous materials with likely slit-like pores near the maximum relative pressure. The isotherms were used to calculate the specific surface area, using the BET method (S_BET_–multipoint), and some textural properties based on the Barrett, Joyner, and Halenda (BJH) model [61] (Table 3). S_BET_ is similar for the three photocatalysts as well as the monolayer adsorption volume (V_m_), whereas the BET C constant is similar for Cu/TiO_2_ and Cr/TiO_2_ photocatalysts and lower for V//TiO_2_, which suggests a weaker interaction between N_2_ and the photocatalyst. S_BET_ values are similar to reported values, e.g., for Cu(0.5–10%)/TiO_2_-P25) ca. 50 m^2^·g^−1^ [30]. Adsorption below a relative pressure ca. 0.07 fits a monolayer adsorption model. Other than the different BET C constants for {Cu, Cr} and V impregnated photocatalysts, the rest of textural properties show similar values. The negative value of the t-plot micropore reinforces the hypothesis of these being mesoporous materials, which is also supported by comparison of their S_BET_ and t-plot external surface areas (Table 3). Pore size distribution (PSD) was determined from the corresponding isotherms using Barrett, Joyner, and Halenda (BJH) and Dollimore–Heal (D–H) models to calculate differential specific pore volume vs. pore width distribution (Figure 7A and Figure S10).

The three M(0.1%)/TiO_2_ photocatalysts showed similar results, a narrow PSD centered at ca. 25 Å, in the lower limit of mesopores, i.e., 2 nm. BJH and D–H models predict the same PSD as shown in the inset of Figure 7A. It was also observed that PSDs using adsorption and desorption data were fully coincident with BJH and D–H models (Figure S10).

PSDs were also calculated by the non-local density functional theory method (NLDFT) [62]. The 2D-NLDFT model (N_2_-Carbon Finite Pores, Aspect Ratio 6, Standard Slit) fitted very well the model isotherms to the experimental ones (Figure S11); the corresponding PSDs for the three impregnated photocatalysts covered a range from 2 to 10 nm (Figure 7B).

Minor differences have been found in the fractal dimension D_S_ (see Section 2.3 and Table 3) between the metal impregnated photocatalysts, Cu(0.1%) 2.542, Cr(0.1%) 2.535, and V(0.1%) 2.523, respectively, the former showing a slightly higher surface roughness (Figures S12–S14 in the Supporting Information).

## 4. Photodegradation of Phenol under Vis and UV Light Irradiation

**Figure 8:**
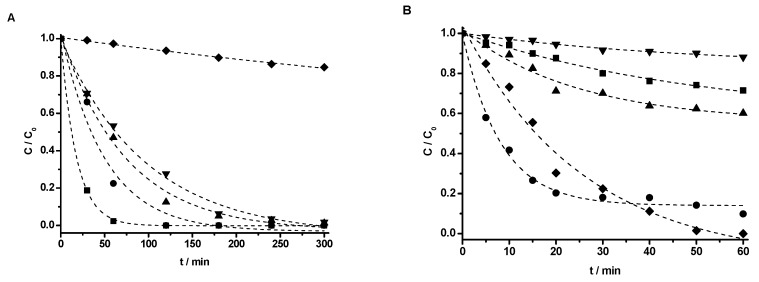
Photodegradation of phenol over Cu(X%)/TiO_2_ under (**A**) Vis (*λ*_exc_ > 366 nm) irradiation, (**B**) UV (*λ*_exc_ = 255 nm) irradiation; HPLC–UV detection (*λ* = 270 nm). X%: 0% (♦), 0.1% (■), 0.3% (●), 0.5% (▲), 1% (▼). [Phenol]0 = 50 mg·L^−1^, [M(X%)/TiO_2_]0 = 1.0 g·L^−1^, natural pH, T = 298.0 K. Dotted lines show the corresponding first order kinetic fits.

The photocatalytic activity of M(X%)/TiO_2_ samples was tested by monitoring phenol degradation in aqueous solution under NUV-Vis (*λ*_exc_ > 366 nm) and UV (*λ*_exc_ = 254 nm) irradiation. The experimental data were in all cases adequately fit by a first order kinetic model (C = C_0_·e^–k·t^), and the corresponding apparent first order rate constants are collected in Table 4. The rate constants obtained by UV-Vis spectrophotometry and HPLC, with UV detection, are comparable, and the small differences observed follow the same pattern. The effect of impregnation with transition metal ions at different concentrations on phenol removal efficiency is displayed in Figure 8 for Cu, as a prototypical example, and all other effects are shown in Figures S15–S24 in the Supplementary Information.

Minor or no changes were found in the kinetics and efficiencies of the process upon three repeated cycles of photodegradation using the same batches of photocatalysts.

Phenol photocatalyzed degradation under UV light irradiation is, in general, faster using non-impregnated TiO_2_-P25 (Figure 8B and Table 4). Though for 0.5% Cu the process is faster than for non-impregnated TiO_2_-P25 in the first 30 min, the efficiency of the process with 0.5% Cu is lower, not reaching full disappearance of phenol. Similar to previous studies, there is an optimum dopant concentration [30,54], here clearly 0.3% for Cu, 0.1% for V, and 0.5% for Cr (Figure 8B and Table 4). Limited phenol removal, measured as the loss of absorbance of the reacting mixture at 270 nm, was found using metal impregnated TiO_2_-P25 photocatalysts under UV irradiation, usually lower than 30% (Figure 8B and Figures S19–S24 in the Supplementary Information).

All metal-impregnated photocatalysts showed phenol removals much faster and efficient than the standard TiO_2_-P25 under NUV-Vis light irradiation. Figure 8A shows complete phenol degradation under NUV-Vis light irradiation using TiO_2_-P25 coated with Cu, much faster using Cu(0.1%)/TiO_2_-P25 (half-life ca. 15 min) than Cu(1%)/TiO_2_-P25 (half-life ca. 43 min). This time the lowest metal content, 0.1%, showed the best result, both in terms of rate and efficiency (understood as reaction extent after 300 min). A similar behavior was described in the photocatalyzed degradation of Malachite Green, as here the fastest photodegradation rate was found for non-impregnated TiO_2_ under UV radiation, whereas it was the slowest under Vis and direct sunlight irradiation. The maximum photodegradation rate was found at Cu(1.71%)–TiO_2_ [49] under Vis irradiation. A similar behavior was also observed in the NUV-Vis photodegradation of methyl orange, maximum at Cu(1%)–TiO_2_ [63].

The photocatalytic efficiency for V/TiO_2_-P25 photocatalysts, under NUV-Vis irradiation, was 99% (59%) after 175 min (300 min) for V(0.1%)/TiO_2_-P25 [V(1%)/TiO_2_-P25]. An optimum dopant concentration has been reported for the degradation of Methylene Blue using V doped TiO_2_; [optimum dopant concentration V(0.5%)–TO_2_] [29], for 4-nitrophenol using [V(0.5%)–TiO_2_ ] [34], and for 2-4-dichlorophenol [V(1%, as V^4+^)–TiO_2_] [32].

In the case of Cr, the removal yield reached the highest value of 70% for Cr(0.1%)/TiO_2_ after 300 min under Vis irradiation. The photoactivity of Cr/TiO_2_-P25 is similar under Vis and NUV irradiation and almost independent of the Cr content and slightly higher than with bare TiO_2_-P25, such behavior matches that of previously reported [23,64,65].

Here the differences in reactivity do not come from the crystallite size (Table 1) or from the surface roughness (vide supra), they are similar for the three photocatalysts. In addition to crystalline structure and specific surface area, there are other factors playing a relevant role in the photocatalytic activity of metal impregnated photocatalysts; metal dopants might behave as hole (h^+^) and/or electron (e^−^) traps, therefore changing the electron/pair recombination rate.

The photocatalytic activity of metal impregnated TiO_2_-P25 does not run parallel to the increase of the red-shift of absorption edge and light absorption in the visible light region with dopant concentration. It is well known that beyond an optimum dopant concentration, here M(0.1%)/TiO_2_-P25, the photocatalytic activity decreases [30]. Several reasons can be argued to explain such photoactivity decline after the dopant concentration optimum. First, it might be due to the increase of e^−^/h^+^ pair recombination as dopant concentration increases. A higher concentration shortens the distance (R) between trap sites of photogenerated e^−^/h^+^ pairs and the recombination rate (k_RR_) of charge carriers increases in accordance with the equation:

(7) $$k_{RR}=e^{-\frac{2\cdot R}{a_{0}}}$$

where a_0_ is the radius of the hydrogenic wave function for the charge carrier, i.e., metal dopants become recombination centers as the distance between trapping sites shortens [46]. The low photocatalytic effectiveness of Cr-impregnated TiO_2_-P25 likely due to the short diffusion length of the charge carriers (ca. 0.2 μm) [65], leading to a faster e^−^/h^+^ recombination rate [32,63].

At the optimum surface impregnation concentration there is an efficient separation between photogenerated h^+^ and e^−^. The space charge region extends and the surface barrier for recombination is maximum, whereas at higher dopant concentration the space charge region narrows, e^−^/h^+^ pairs are produced in the bulk of the photocatalyst, deep trap instead of shallow trap takes place, and volume recombination dominates leading to reduced photocatalytic activity.

**Figure 9:**
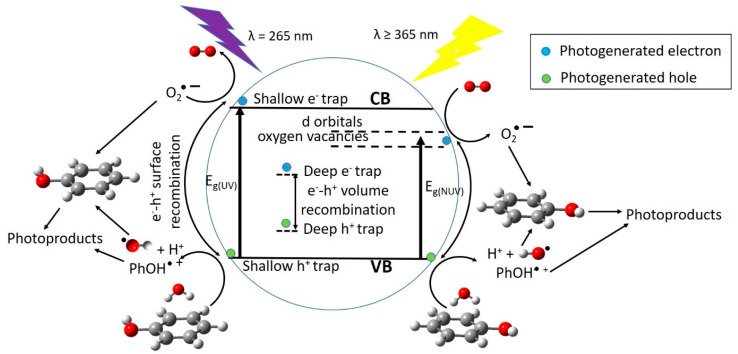
Processes involved in the photocatalyzed degradation of phenol with M(%)/TiO_2_-P25 under UV and near UV-Vis light (NUV-Vis) irradiation.

Second, a decrease in specific area with concentration means reduction of photoactivity. Third, we hypothesized that the shading effect due to the higher surface coverage as the dopand concentration increases, which also means minor contact area between phenol and TiO_2_-P25 [32]. Fourth, the metal ion can bind to surface hydroxyl groups thus reducing the availability of –OH groups to be converted into HO radicals. The increased photocatalytic activity of Cu and V-impregnated TiO_2_-P25 in the degradation of phenol, under NUV-Vis light irradiation, points to different mechanism of photoactive enhancement under UV and NUV-Vis radiation. In the latter, photoelectrons are transferred from impregnated TiO_2_-P25 VB to Cu or V 3d-orbitals lying just below of the CB, then migrate to form O_2_^–^ radicals, whereas holes migrate to the surface, react with HO^–^ rendering HO radicals, then the so-formed radicals are able to initiate the degradation of the adsorbed phenol molecules (Figure 9). Metal ions with charge different than Ti^4+^ could produce oxygen vacancies in the lattice with energy levels below the TiO_2_-P25 CB (Figure 9), allowing visible light harvesting, acting as active sites for adsorbed water dissociation and capturing holes to diminish electron-hole recombination, thus enhancing the photocatalytic activity [35,66,67].

Photocatalytic activity, among other factors, is not only dependent on the photogenerated charge carriers trapping, efficient detrap to the surface should also occur. Metal dopants can act as h^+^ traps (M^n+^ + h_VB_^+^ → M^(n+1)+^) and/or e^−^ traps (M^n+^ + e_CB_^–^ → M^(n–1)+^), the energy levels of M^n+^/M^(n+1)+^ and M^n+^/M^(n–1)+^ lying above VB and below CB of TiO_2_-P25, respectively [47]. The stability of M^(n+1)+^ and M^(n–1)+^ depends on the change on the electronic configuration relative to the initial electronic configuration of M^n+^, for instance e^−^ trap, better than h^+^ trap should occur for Cu^2+^, in this way d-orbitals become completely filled. V^5+^ and Cr^3+^ should act as hole traps, whereas, for example, it is accepted that V^4+^ serve both as h^+^ and e^−^ trap [47]. Then M^(n–1)+^ can transfer the trapped electron to the TiO_2_-P25 lattice, and from there to adsorbed O_2_ molecules to yield O_2_^–^, or trap a VB h^+^ turning back to its stable electronic configuration. On the other hand, M^(n+1)+^ can transfer its additional positive charge either to adsorbed HO^–^, forming the reactive HO radical, or to adsorbed phenol molecules (Figure 9).

In summary, all the studied impregnated photocatalysts were less efficient under UV irradiation (extent of reaction after 60 min) than the standard TiO_2_-P25, where photoactivity follows the order: TiO_2_-P25 >> Cu > V > Cr, irrespective of the metal percentage in the photocatalyst; whereas under NUV-Vis irradiation, phenol photocatalyzed degradation over M(%)/TiO_2_ composites is faster and more efficient, irrespective of the dopant content, than with TiO_2_-P25, the reactivity order being Cu > V >> Cr > TiO_2_-P25 (extent of reaction after 300 min).

### Total Organic Carbon

**Figure 10:**
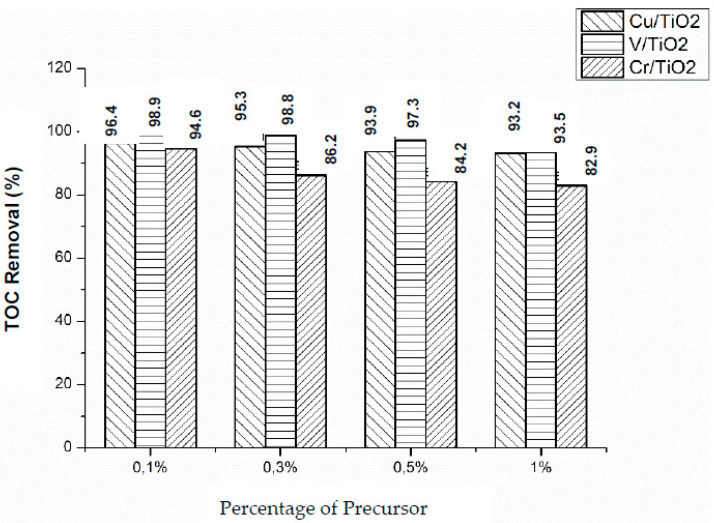
Total organic carbon (TOC) removal during the photocatalytic degradation of phenol over M(X%)/TiO_2_ after 300 min of NUV-Vis light irradiation. [Phenol]0 = 50 mg·L^−1^, [M(X%)/TiO_2_]0 = 1.0 g·L^−1^, natural pH, and T = 298.0 K.

**Figure 11:**
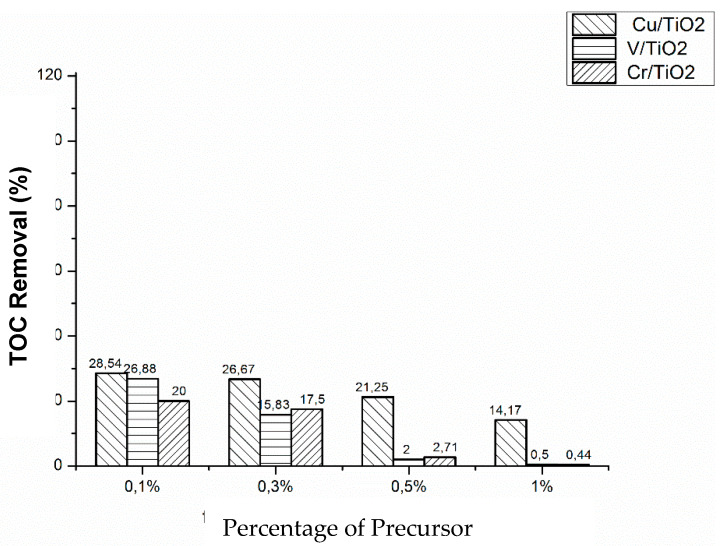
TOC removal during the during photocatalytic degradation of phenol over M(X%)/TiO_2_ after 60 min under UV irradiation. [Phenol]0 = 50 mg·L^−1^, [M(X%)/TiO_2_]0 = 1.0 g·L^−1^, natural pH, and T = 298.0 K.

TOC measurements were carried out to determine the degree of mineralization reached, under both Vis and UV light irradiation. Figure 10 shows the obtained results for the three photocatalysts (X = 0.1%, 0.3%, 0.5%, and 1%) under Vis irradiation. V/TiO_2_ was the most efficient photocatalyst for TOC removal, with the maximum removal observed for the lowest metal content (0.1%), decreasing as the metal percentage increases, in accordance with kinetic results. On the other hand, Cr/TiO_2_ photocatalysts are the less efficient in terms of TOC removal. Comparison between kinetic observations and TOC results suggests that phenol disappearance is faster than mineralization, some organic intermediate photoproducts remain in solution, and the amount depends on the impregnation metal and its concentration. Similar behavior was observed with UV light irradiation (Figure 11), although much lower TOC removals were obtained with the three metal impregnated photocatalysts, which is consistent with kinetic runs (vide supra). The most efficient, in terms of TOC removal, was Cu(0.1%)/TiO_2_.

Phenol photocatalyzed degradation over M/TiO_2_ (M: Cu, Cr, and V), measured either as phenol disappearance or as TOC removal, is higher under NUV-Vis light than under UV irradiation. We have previously found very high efficiencies of NUV-Vis photocatalytic TOC removal using photocatalysts doped with Cu [25]. Thinking in pollution abatement, although slower, photodegradation is more effective under NUV-Vis, and in economic terms longer time using NUV-Vis radiation counterweight the costs of using UV irradiation sources.

## 5. Reaction Pathways for Photocatalyzed Degradation

Photocatalytic degradation of phenol is a complex multi-stage process. The photocatalytic process with M–TiO_2_ (M = Cu, V, and Cr) is energetically favourable for the decomposition of phenolic compounds with respect to the process with the standard TiO_2_–P25. Two types of oxidizing species, i.e., the radical hydroxyl HO and superoxide O_2_^–^ are involved in the transformation of the aromatic compounds [15]. The lifetime of the intermediates formed at different stages of the reaction is short, as they undergo further fast catalytic oxidation.

**Figure 12:**
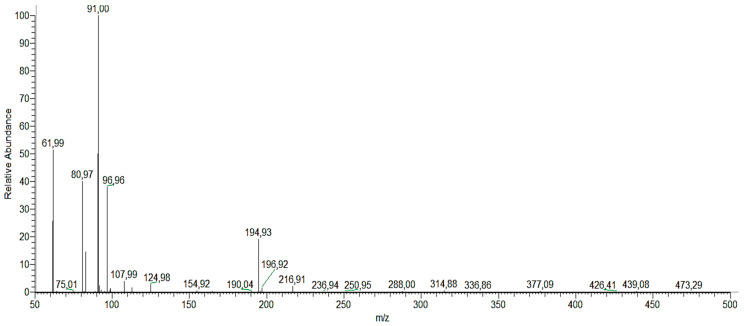
HPLC–MS mass spectra of phenol photoproducts.

We determined the different reaction intermediates using HPLC–MS. Figure 12 shows a typical HPLC chromatogram. The photoproducts found under both UV and Vis light are summarized in Table 5. Identification of the intermediate products is based on the obtained MS and compared to those in databases. The main intermediates identified in this way were: **(1)** catechol, resorcinol and/or hydroquinone, **(2)** phloroglucinol, **(3)** cyclohex-2-ene-1, 2, 4, 5-tetraol, **(4)** (Z)-penta-2,4-dienoic acid, **(5)** carbonic acid, **(6)** (Z)-penta-2,4-dienal, **(7)** juglone, **(8)** 9H-xanthen-9-one, **(9)** 3-hydroxy-2-naphthoic acid, **(10)** 3-Hydroxy-2-naphthoate, and **(11)** (2E)-3-(2-formylphenyl) acrylic acid. Degradation pathways into smaller molecules are proposed and also routes leading to heavier transformation products.

**Figure 13:**
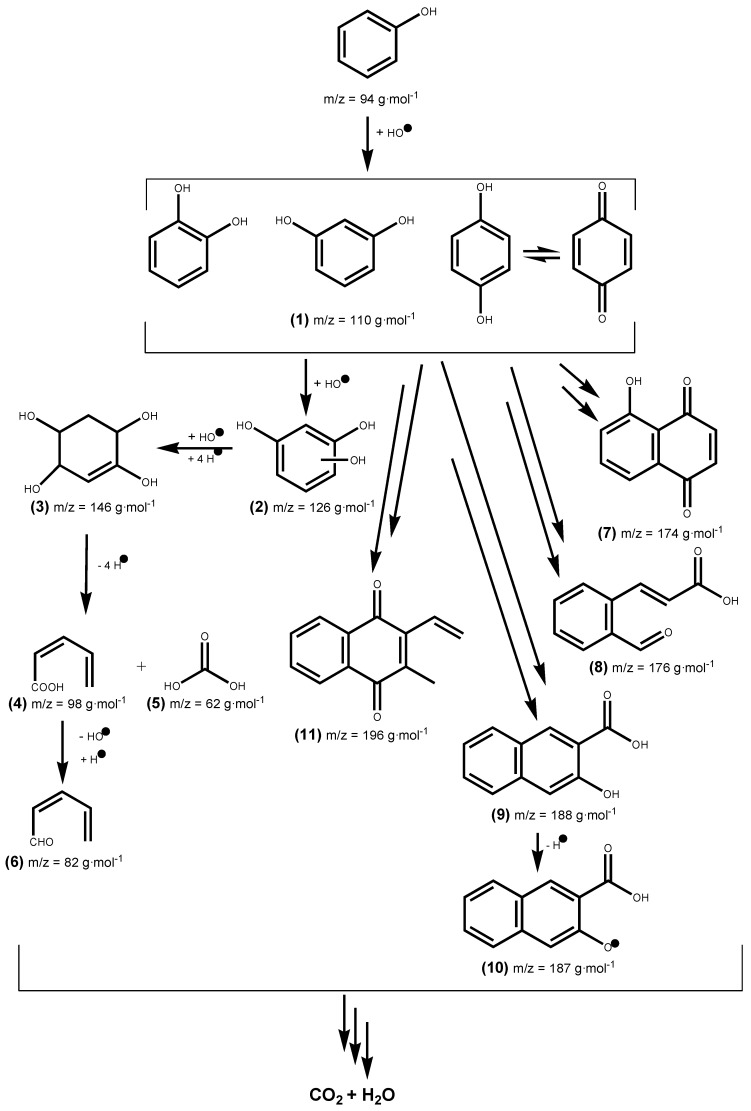
Proposed reaction pathways for the photocatalytic degradation of phenol over M/TiO_2_ (M = Cu, V, and Cr) under UV and visible irradiation. m/z ratios are rounded here, exact values are given in Table 5.

Phenol photocatalyzed degradation over titania-coated metal composites under UV and Vis light irradiation is described by the reaction mechanism shown in Figure 13. Photocatalyzed phenol degradation proceeds through the widely accepted mechanisms of electrophilic attack promoted by HO or h^+^ oxidation of the adsorbed phenol onto the photocatalyst surface [66]. Though a deep understanding of the surface processes taking place is out of the scope of this article, in support to the previous statement, we have observed that the presence of *iso*-Propanol as HO scavenger or EDTA as h^+^ scavengers inhibit the process largely or completely.

Phenol oxidation occurs by hydroxylation to yield dihydroxylbenzenes (**1**) (catechol, resorcinol, and/or hydroquinone) [67,68]. Further hydroxylation produces phloroglucinol (**2**) and likely other trihydroxybenzenes. The hydroxylation of the former with addition of hydrogen gives (**3**) which leads to intermediates (**4**) and (**5**) by ring opening via C–C bond breaking, then dehydroxylation of (**4**) and hydrogen addition leads to (**6**). Several radical species are formed in phenol photodegradation [69,70,71,72] that can react between them to give the other intermediates (**7**–**11**) found in this work.

## 6. Photodegradation and Energetic Efficiency of the Process

Light scattering by the suspended catalyst particles and the characteristics of the surface contribute to reduce the photodegradation quantum yield (Φ_photodegradation_) and the photonic efficiency of the process (ξ) [73].

The photocatalysis quantum yield (Φ_Photocatalysis_) is the ratio of moles of reactant consumed per Einstein absorbed by the photocatalyst [74]. It can be calculated as [75,76,77]:

(8) $$\Phi_{photodegradation}=\frac{k_{app}}{2.303\cdot I_{\lambda }\cdot \varepsilon_{\lambda }\cdot l}$$

where Φ_photodegradation_ is the photodegradation quantum yield, k_app_ is the apparent pseudo first order rate constant, *I_λ_* (Einstein·L^−1^·s^−1^) is the light intensity at wavelength *λ*, ε*_λ_* (cm^−1^·mol·dm^–3^) is the molar absorptivity at *λ*, and l is the path length of the photoreactor (cm). The so-obtained Φ_photodegradation_ are shown in Table 6. Both for UV and UVA-Vis lamps, Φ_photodegradation_ (Cu) > Φ_photodegradation_ (V) > Φ_photodegradation_ (Cr), with values higher than 1 for Cu and V, pointing to the existence of secondary processes, that inflate the efficiency of the incident photons.

Photodegradation of organic micropollutants in aqueous solution is energy demanding, and an energy efficiency parameter (E_EO_) can be defined to analyse it, as the kWh of energy required to reduce the pollutant concentration per volume and time unit (kW·L^−1^·s^−1^) [78], expressed as:

(9) $$E_{E_{0}}=\frac{38.4\cdot P}{V\cdot k_{app}}$$

where P is the electric power consumed by the lamp (kW), V is the volume (L) of solution and k_app_ are the apparent photodegradation rate constants from Table 4. The values thus obtained for E_EO_, compiled in Table 6, are lower with 254 nm than with 366 nm, showing that E_EO_ is far more favourable with UV lamps. The efficiency order varies, both for UV and UVA-Vis lamps, in the order E_EO_ (Cu) < E_EO_ (V) < E_EO_ (Cr).

## 7. Conclusions

The photocatalyzed degradation of phenol, as a model pollutant, in aqueous solution over titania-coated metal (M = Cu, Cr, and V) composites under visible (*λ*_exc_ > 366 nm) and UV (*λ*_exc_ = 255 nm) irradiation is described. Metal surface impregnated photocatalyts were synthesized using the wet impregnation method and characterized by X-ray diffraction (XRD), X-ray fluorescence (XRF), UV-Vis diffuse reflectance spectroscopy (UV-Vis DRS), and surface area (BET). No oxides phases of the metal dopants were found. Analysis of XRD peak broadening, in terms of the uniform deformation model (UDM), points to the existence of tensile strain in Cu-impregnated TiO_2_-P25, whereas compressive in Cr- and V-impregnated TiO_2_-P25.

UV-Vis DRS measurements clearly show the dependence of the band gap on the synthetic method. Raman spectra showed an enhancement of the crystallinity of the anatase phase by impregnation mainly with Cu and V cations. Red-shifts were also observed on the E_g_ (140 cm^−1^) peak (ΔRaman-shift = 2.8–4.4 cm^−1^) in M(0.1%)/TiO_2_ samples, especially for Cu(0.1%)/TiO_2_. Experimental evidences suggest at least a surface metal-linkage.

Type IV N_2_ adsorption–desorption isotherms were found, with a small H3 loop near the maximum relative pressure. S_BET_ was similar for the three M(0.1%)/TiO_2_ photocatalysts, ca. 45 m^2^·g^−1^. Pore size distribution using BJH, D–H, and NLDFT models suggest those photocatalysts are mesoporous with a narrow pore distribution centered at ca. 25 Å, which is in agreement with the rest of textural data. PSDs using BJH and D–H models are fully coincident, and the same is observed using adsorption and desorption branches. Metal surface impregnated photocatalysts show similar surface roughness.

Their photocatalytic activity was followed by UV-Vis spectroscopy and HPLC–UV. A first-order equation was used to fit kinetic data. Similar results are obtained using UV-Vis spectroscopy and HPLC monitoring. Fastest phenol degradation was obtained with M(0.1%)/TiO_2_ the order being Cu > V >> Cr > TiO_2_/P25 under Vis radiation, whereas reactivity was TiO_2_/P25 >> Cu > V > Cr under UV radiation.

The degree of mineralization was calculated in terms of TOC removal efficiency, and we were able to achieve more than 95% disappearance of the total organic carbon using visible light and less than 30% when treated with UV light.

Products and intermediate organic photoproducts were identified by HPLC–MS spectrometry, and the corresponding kinetic mechanism proposed.

Finally, the energetic efficiency of the process was analysed for M (0.1%), showing that UV lamps are far superior to UVA lamps, and that the efficiency of the surface impregnated catalyst varies in the order E_EO_ (Cu) < E_EO_ (V) < E_EO_ (Cr).

## Figures and Tables

**Table 1:** Position of selected diffraction peaks, crystallite size (τ), microstrain (ε), and anatase mass fraction of Cu, Cr, and V surface-impregnated TiO_2_-P25 photocatalysts.

		Cu(0.1%)/TiO_2_-P25		Cr(0.1%)/TiO_2_-P25		V(0.1%)/TiO_2_-P25	
Phase	(h k l)	2Ө/^0^	Τ ^a^/nm	2Ө/^0^	Τ ^a^/nm	2Ө/^0^	Τ ^a^/nm
Anatase	(1 0 1)	25.399	22.4	25.362	22.4	25.404	22.4
	(0 0 4)	37.923	21.9	37.894	24.4	37.92	24.4
	(2 0 0)	48.152	17.9	48.124	23.9	48.145	21.0
	(2 1 5)	62.834	20.0	62.824	27.1	62.819	26.3
	W–H ^b^	τ/nm	27.4		20.2		20.8
		Strain (ε)	8.5 × 10^–4^	−8.0 × 10^–4 c^		−5.3 × 10^–4 c^	
	SSP ^d^	τ/nm	12.9		12.9		12.1
		Strain (ε) ^c^	0.011	0.011		−0.005 ^c^	
Rutile	(1 0 1)	27.511	36.8	27.524	32.4	27.516	40.4
	(1 0 1)	36.185	30.6	36.162	39.4	36.163	31.8
	(1 1 1)	41.134	35.0	41.305	32.3	41.387	46.7
	W–H ^b^	τ/nm	42.8		31.9		32.6
		Strain (ε) ^c^	6.8 × 10^–4^	−2.8 × 10^–4 c^		−5.5 × 10^–4 c^	
	SSP ^d^	τ/nm	21.4		16.3		19.7
		Strain (ε)	0.009	−0.006 ^c^		−0.004 ^c^	
*Anatase mass fraction (%)* ^e^			81.5	79.4		78.6	

^a^ Scherrer equation [17]; ^b^ Williamson–Hall equation (W–H) (Figures S2–S4) [18]; ^c^ From the negative value of either the W–H equation slope or the size–strain plot (SSP) intercept (Figures S2–S4); ^d^ Size–Strain plot (Figures S5–S7); ^e^ Calculated using Spurr and Myers equation (see Section 2.3) [16].

**Table 2:** Band gap values obtained for the metal surface-impregnated TiO_2_ photocatalysts. The entries in bold were obtained in this work (M(X%)/TiO_2_-P25).

% M/TiO_2_	V	Cr	Cu
0	**3.31**; 3.26 [34,35]	**3.31**; 3.18 [23]	**3.30**
0.02	2.92 [35]		
0.06	2.72 [35]		
0.1	**3.26**; 2.78 [34,35]	**3.30**; 3.16 [23]	**3.29**
0.2			3.0 [48]
0.3	**3.29**	**2.05**	**3.50**
0.5	**3.26**	**2.30**; 3.06 [23]	**2.44**; 3.14 [23]
0.88			2.72 [49]
1.0	**3.21**	**2.44**, 3.04 [23]	**2.07**; 3.22 [23]

**Table 3:** BET parameters and textural properties of V(0.1%)/TiO_2_, Cu(0.1%)/TiO_2_ and Cr(0.1%)/TiO_2_ photocatalysts measured by N_2_ adsorption–desorption. TiO_2_-P25 BET surface area = 51 (m^2^·g^−1^) [30].

Photocatalyst		V(0.1%)/TiO_2_			Cu(0.1%)/TiO_2_			Cr(0.1%)/TiO_2_		
BET	S_BET_/m^2^·g^−1^	44.38 ± 0.07			46.92 ± 0.04			47.57 ± 0.05		
	Constant C	72			103			107		
	V_m_ (monolayer adsorption volume)/cm^3^·g^−1^	10.2			10.8			10.9		
Parameter		Surface area (m^2^·g^−1^)	Pore volume (cm^3^·g^−1^)	Average pore width (4V/Å)	Surface area (m^2^·g^−1^)	Pore volume (cm^3^·g^−1^)	Average pore width (4V/Å)	Surface area (m^2^·g^−1^)	Pore volume (cm^3^·g^−1^)	Average pore width (4V/Å)
t-plot external surface area		46.84			46.58			47.04		
t-plot micropore volume			−0.001753			-0.000222			–0.000109	
BJH adsorption		40.693 ^a^	0.061463 ^b^	60.416	43.234	0.068866	63.716	44.422	0.073953	66.592
BJH desorption		40.754 ^a^	0.061655 ^b^	60.514	43.659	0.069223	63.422	44.922	0.074221	66.089
D–H adsorption		40.584 ^a^		60.425	43.131		63.712	44.321		66.583
D–H desorption		40.575 ^a^		60.543	43.555		63.405	44.820		66.067
*Maximum pore volume* at p/p°/cm³/g (STP)			0.17713625	Median pore width		0.17706067	Median pore width		0.17714428	Median pore width
			*0.01802*	7.687 Å		*0.01936*	7.759 Å		*0.01965*	7.787 Å
Average particle size/Å		1352			1279			1261		
Fractal dimension (D_S_)		2.523			2.542			2.535		

^a^ Cumulative surface area of pores between 1.7 and 300 nm in diameter; ^b^ Cumulative pore volume of pores between 1.7 and 300 nm in diameter.

**Table 4:** Apparent degradation rate constants obtained in the photocatalyzed degradation of phenol over M(%X)/TiO_2_ composites under Vis (*λ*_exc_ > 366 nm) and UV (*λ*_exc_ = 255 nm) irradiation. [Phenol] 0 = 50 mg·L^−1^, [M(X%)/TiO_2_]0 = 1.0 g·L^−1^, natural pH, T = 298.0 K.

Catalyst	Detection ^a^/Irradiation ^b^		(k ± σ_k_)·10^4^/min^−1^	(k±σ_k_)·10^4^/min^−1^	
**TiO_2_-P25**	**HPLC/UV**	**765 ± 96**	HPLC/Vis	6.0 ± 0.2	
M(X%)/TiO_2_		(k ± σ_k_)·10^4^/min^−1^			
		0.1%	0.3%	0.5%	1.0%
Cu	S/UV	50 ± 3	328 ± 45	80 ± 6	16 ± 7
	HPLC/UV	58 ± 4	324 ± 53	87 ± 10	21 ± 2
	S/Vis	435 ± 50	252 ± 40	257 ± 29	183 ± 15
	HPLC/Vis	493 ± 51	394 ± 103	151 ± 7	140 ± 8
V	S/UV	44 ± 5	41 ± 4	35 ± 2	31 ± 3
	HPLC–UV	50 ± 5	49 ± 3	45 ± 3	36 ± 3
	S/Vis	233 ± 13	211 ± 21	89 ±4	34 ± 2
	HPLC/Vis	244 ± 14	193 ± 13	131 ± 7	49 ± 3
Cr	S/UV	27 ± 3	32 ± 2	52 ± 4	37 ± 3
	HPLC–UV	24 ± 3	28 ± 1	46 ± 3	35 ± 2
	S/Vis	33 ± 5	25 ± 3	22 ± 1	19 ± 1
	HPLC/Vis	34 ± 5	23 ± 2	24 ± 1	21 ± 1

**Table 5:** HPLC–MS data for phenol photoproducts in the photocatalyzed decomposition of phenol over titania-coated metal composites under UV and Vis irradiation.

Photoproducts	(M−H)^−^ (m/z)	t_R_ (min)
**(1)** catechol, resorcinol and/or hydroquinone	109.967	1.7
**(2)** phloroglucinol	125.11	1.47
**(3)** cyclohex-2-ene-1, 2, 4, 5-tetraol	145.141	5.48
**(4)** (Z)-penta-2,4-dienoic acid	96.96	1.68
**(5)** carbonic acid	61.988	1.48
**(6)** (Z)-penta-2,4-dienal	80.974	1.38
**(7)** juglone	173.15	7.9
**(8)** (2E)-3-(2-formylphenyl) acrylic acid	194.927	1.51
**(9)** 3-hydroxy-2-naphthoic acid	187.101	7.9
**(10)** 3-hydroxy-2-naphthoate	186.172	6.95
**(11)** 9H-xanthen-9-one	174.96	0.97

**Table 6:** Photodegradation quantum yields (Φphotodegradation) and energy efficiency (E_EO_) for the photocatalyzed degradation of phenol over (0.1% M)/TiO_2_ composites under Vis (*λ*_exc_ > 366 nm) and UV (*λ*_exc_ = 255 nm) irradiation. [Phenol]0 = 50 mg·L^−1^, [0.1% M)/TiO_2_]0 = 1.0 g·L^−1^, natural pH, and T = 298.0 K.

Lamp	(0.1% M)/TiO_2_	Φ_photodegradation_	E_EO_/kW·L^−1^·s^−1^
UV (254 nm)	Cu	1.17	6400
	V	1.01	7385
	Cr	0.56	13395
UVA-Vis (*λ*_exc_ >366 nm)	Cu	2.81	37403
	V	1.46	72000
	Cr	0.20	514286

## Supplementary Materials

The following are available online at https://www.mdpi.com/2079-4991/10/5/996/s1, Figure S1: SEM images of (a, d) Cr(0.1%)/TiO_2_, (b,e) Cu(0.1%)/TiO_2_ and (c,f) V(0.1%)/TiO_2_, Figure S2: Williamson-Hall plot for Cu(0.1%)/TiO_2_-P25, anatase and rutile, Figure S3: Williamson-Hall plot for Cr(0.1%)/TiO_2_-P25, anatase and rutile, Figure S4: Williamson-Hall plot for V(0.1%)/TiO_2_-P25, anatase and rutile, Figure S5: Size-Strain plot for Cu(0.1%)/TiO_2_-P25, anatase and rutile, Figure S6: Size-Strain plot for Cr(0.1%)/TiO_2_-P25, anatase and rutile, Figure S7: Size-Strain plot for V(0.1%)/TiO_2_-P25, anatase and rutile, Figure S8: N2 adsorption-desorption isotherm of V(0.1%)/TiO_2_-P25 photocatalyst, Figure S9: N2 adsorption-desorption isotherm of Cr(0.1%)/TiO_2_-P25 photocatalyst, Figure S10: Differential specific pore volume vs. pore width distribution for Cu(0.1%)/TiO_2_-P25 photocatalyst using the BJH model. Inset: using the D-H model, Figure S11: Comparison between observed and calculated isotherm of Cu(0.1%)/TiO_2_-P25 photocatalyst, Figure S12: Frenkel-Halsey-Hill fractal analysis of the Cu(0.1%)/TiO_2_-P25 isotherm, Figure S13: Frenkel-Halsey-Hill fractal analysis of the Cr(0.1%)/TiO_2_-P25 isotherm, Figure S14: Frenkel-Halsey-Hill fractal analysis of the V(0.1%)/TiO_2_-P25 isotherm, Figure S15: Photodegradation of phenol over Cu(X%)/TiO_2_ under NUV-Vis irradiation, Figure S16: Photodegradation of phenol over Cu(X%)/TiO_2_ under UV irradiation, Figure S17: Photodegradation of phenol over V(X%)/TiO_2_ under NUV-Vis irradiation, Figure S18: Photodegradation of phenol over V(X%)/TiO_2_ under NUV-Vis irradiation, Figure S19: Photodegradation of phenol over V(X%)/TiO_2_ under UV irradiation, Figure S20: Photodegradation of phenol over V(X%)/TiO_2_ under UV irradiation, Figure S21: Photodegradation of phenol over Cr(X%)/TiO_2_ under NUV-Vis irradiation, Figure S22: Photodegradation of phenol over Cr(X%)/TiO_2_ under NUV-Vis irradiation, Figure S23: Photodegradation of phenol over Cr(X%)/TiO_2_ under UV irradiation, Figure S24: Photodegradation of phenol over Cr(X%)/TiO_2_ under UV irradiation, Table S1: Scherrer crystallite size, anatase mass fraction, main diffraction peaks and indexation for XRD of 0.1% Cu, Cr and V doped TiO_2_-P25 photocatalysts before and after 2 h suspension in water.
