# Erratum: Canle, M., et al. Improved Photocatalyzed Degradation of Phenol, as a Model Pollutant, over Metal-Impregnated Nanosized TiO_2_. *Nanomaterials* 2020, *10*, 996

**DOI:** 10.3390/nano10061109

**Published:** 2020-06-03

**Authors:** S. Belekbir, M. El Azzouzi, A. El Hamidi, L. Rodríguez-Lorenzo, J. Arturo Santaballa, M. Canle

**Affiliations:** 1 Laboratoire de Physico-Chimie des Matériaux et Nanomateriaux, Faculté des Sciences, Université Mohammed V, Avenue Ibn Battouta, Rabat BP 1014, Morocco; belekbirsoukayna@gmail.com (S.B.); elazzouzim@hotmail.com (M.E.A.); adnane_el@gmail.com (A.E.H.); 2 International Iberian Nanotechnology Laboratory, 4715-330 Braga, Portugal; laura.rodriguez-lorenzo@inl.int; 3 Grupo React!, Departamento de Química, Facultade de Ciencias & CICA, Universidade da Coruña, E-15071 A Coruña, Spain; arturo.santaballa@udc.es

The authors wish to make the following corrections to this paper:

