# Supplementary material for: Improved Photocatalyzed Degradation of Phenol, as a Model Pollutant, over Metal-Impregnated Nanosized TiO2
Source: Nanomaterials (Basel). 2020 May 22;10(5):996. doi: 10.3390/nano10050996 (PMC7279559; doi:10.3390/nano10050996)
Supplement: Supplementary file 1 [file nanomaterials-10-00996-s001.pdf]

Supplementary information

Improved Photocatalyzed Degradation of Phenol, as a Model Pollutant, over Metal-Impregnated Nanosized TiO_2

S. Belekbir [1] M. El Azzouzi [1], A. El Hamidi [1], L. Rodríguez-Lorenzo [2], J. Arturo Santaballa [3] and M. Canle [3,*]

1 Laboratoire de Physico-Chimie des Matériaux et Nanomateriaux, Faculté des Sciences, Université Mohammed V, Avenue Ibn Battouta, BP 1014, Agdal, Rabat, Morocco; belekbirsoukayna@gmail.com (S.B.); elazzouzim@hotmail.com (M.E.A.); adnane_el@gmail.com (A.E.H.)

2 International Iberian Nanotechnology Laboratory, 4715-330 Braga, Portugal; laura.rodriguez-lorenzo@inl.int

3 Universidade da Coruña. Grupo React!, Departamento de Química, Facultade de Ciencias & CICA. E-15071 A Coruña, Spain; arturo.santaballa@udc.es

* Correspondence: moises.canle@udc.es

Received: 7 April 2020; Accepted: 18 May 2020; Published: date

Table S1. Scherrer crystallite size, anatase mass fraction, main diffraction peaks and indexation for XRD of 0.1% Cu, Cr and V doped TiO_2-P25 photocatalysts before and after 2 h suspension in water. Anatase and Rutile are shown for reference.

2Θ / ⁰ Anatase	h k l	2Θ / ⁰ 0.1%Cu	0.1%Cu$_{supension}$	0.1%Cr	0.1%Cr$_{supension}$	0.1% V
25.356	(1 0 1)	25.399	25.402	25.362	25.387	25.404
	τ^{a} / nm	22.4	23.0	22.4	21.2	22.4
37.014	(1 0 3)	37.108	37.087	37.054	37.077	37.131
37.847	(0 0 4)	37.923	37.923	37.894	37.930	37.92
48.145	(2 0 0)	48.152	48.184	48.124	48.156	48.145
53.974	(1 0 5)	54.022	54.047	54.022	54.088	54.019
55.186	(2 1 3)	55.194	55.200	55.160	55.147	55.185
62.879	(2 1 5)	62.834	62.838	62.824	62.848	62.819
**2Θ / ⁰ Rutile**	**h k l**	**2Θ / ⁰ 0.1%Cu**	**0.1%Cu$_{supension}$**	**0.1%Cr**	**0.1%Cr$_{supension}$**	**0.1% V**
27.439	(1 1 0)	27.511	27.547	27.524	27.515	27.516
	τ^{a} / nm	36.8	32.4	36.8	35.2	40.4
36.087	(1 0 1)	36.185	36.174	36.162	36.185	36.163
41.247	(1 1 1)	41.314	41.344	41.305	41.330	41.387
54.329	(2 1 1)	54.315	54.401	54.371	54.411	54.442
Anatase mass fraction (%)^{b}		81.5	80.5	79.4	78.9	78.6

a Scherrer equation

b Calculated using Spurr & Myers equation (see section 2.3) (16)

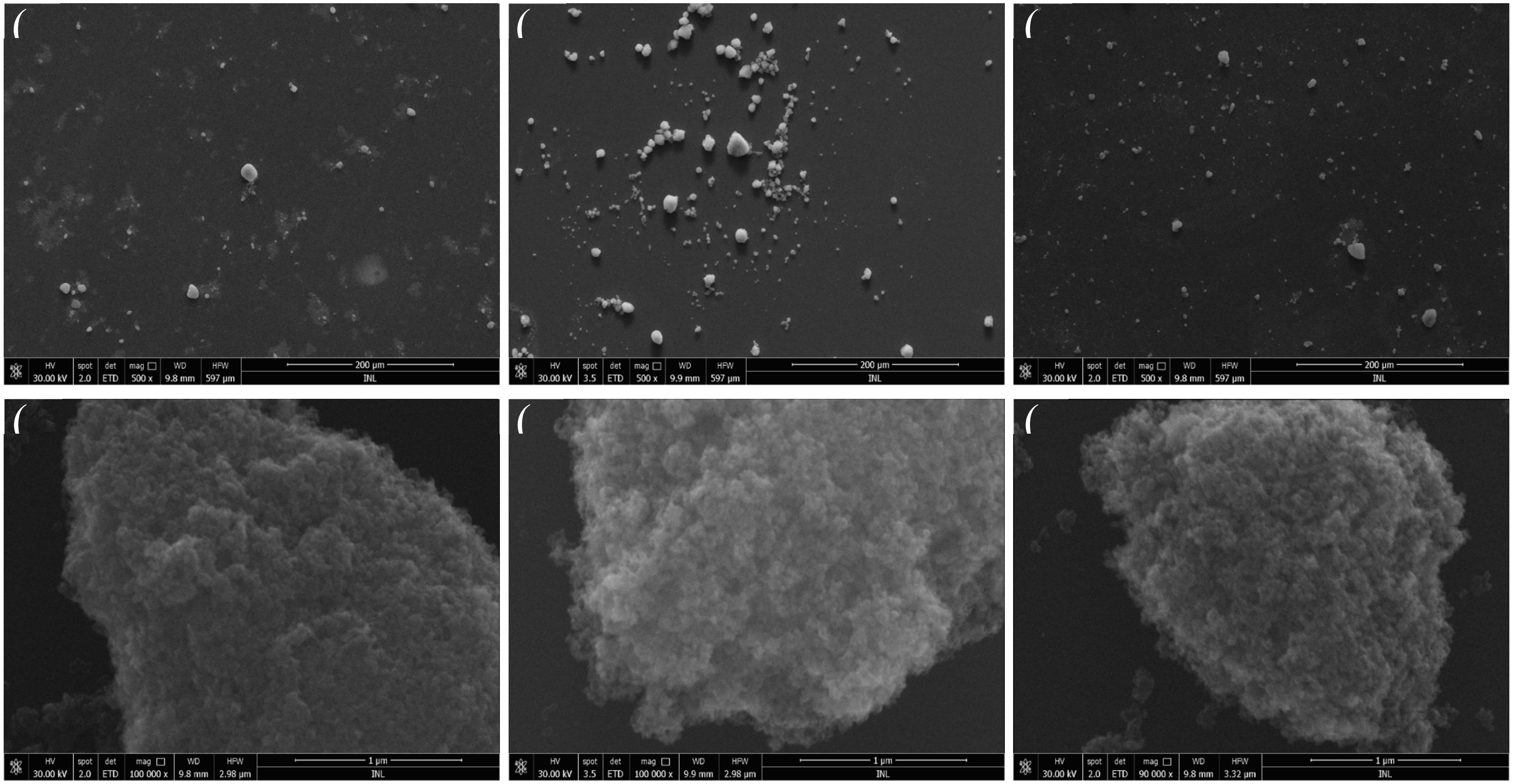

Figure S1. SEM images of (**a**,**d**) Cr(0.1%)/ TiO_2, (**b**,**e**) Cu(0.1%)/ TiO_2 and (**c**,**f**) V(0.1%)/ TiO_2. (**a**), (**b**) and (**c**) show the large agglomerates distribution. Clearly, the highest formation of agglomeration appears on Cu(0.1%)/TiO_2 sample. (**d**), (**e**) and (**f**) show a representative agglomerate at high magnification. These agglomerates are formed for primary TiO_2 nanoparticles with a diameter range of 20 – 35 nm, as measured in TEM.

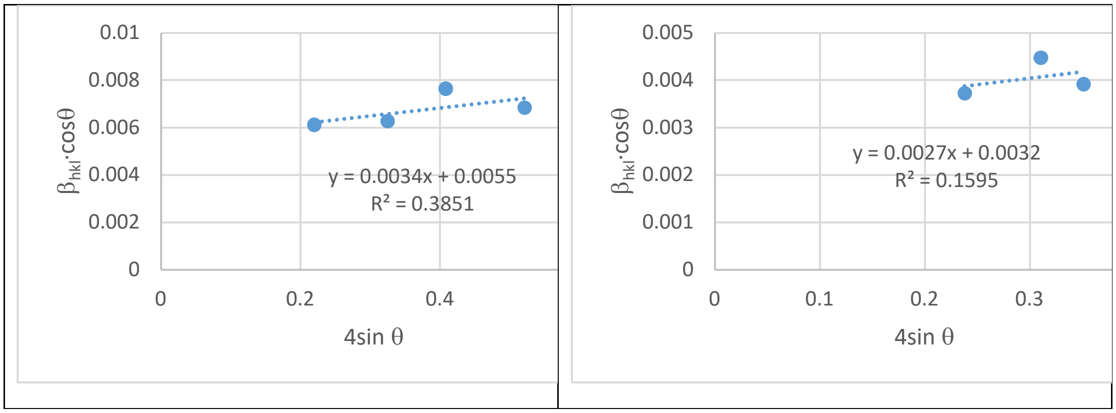

Figure S2. Williamson-Hall plot for Cu(0.1%)/TiO_2-P25, anatase phase (left), rutile phase (right).

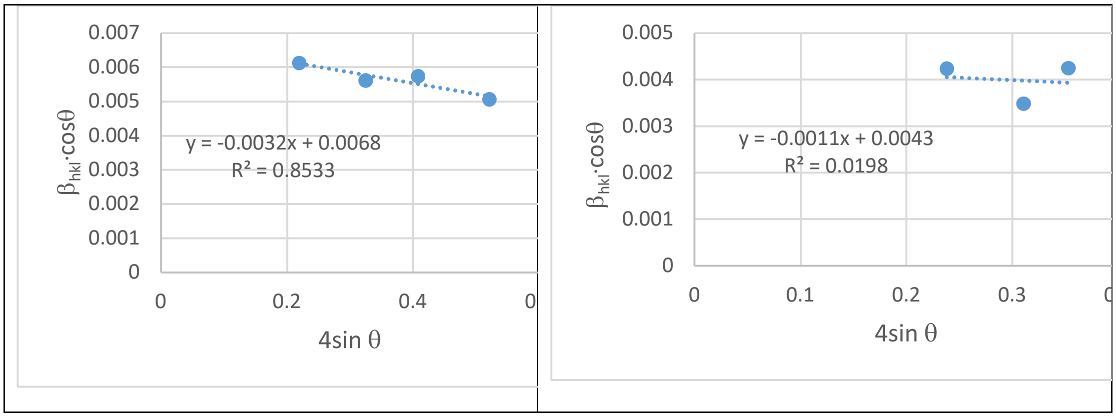

Figure S3. Williamson-Hall plot for Cr(0.1%)/TiO_2-P25, anatase phase (left), rutile phase (right).

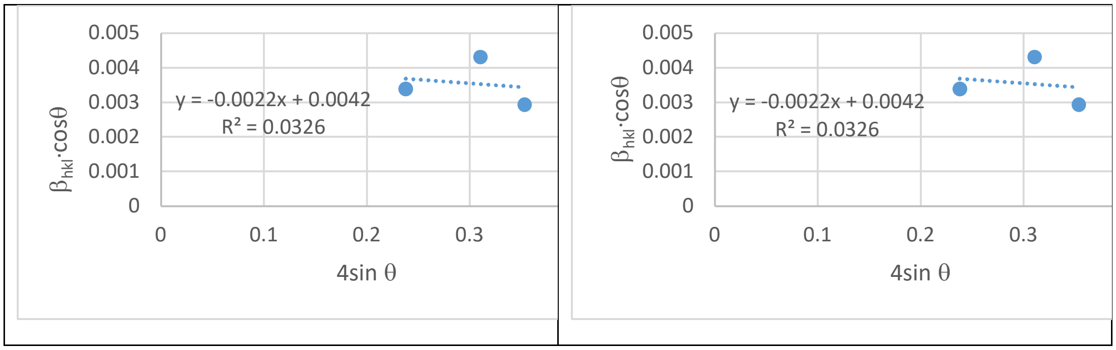

Figure S4. Williamson-Hall plot for V(0.1%)/TiO_2-P25, anatase phase (left), rutile phase (right).

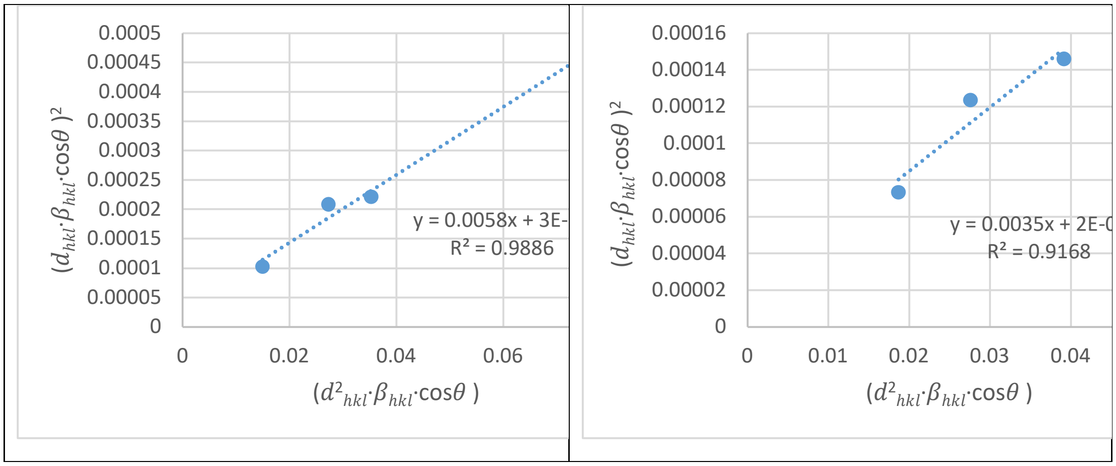

Figure S5. Size-Strain plot for Cu(0.1%)/TiO_2-P25, anatase phase (left), rutile phase (right).

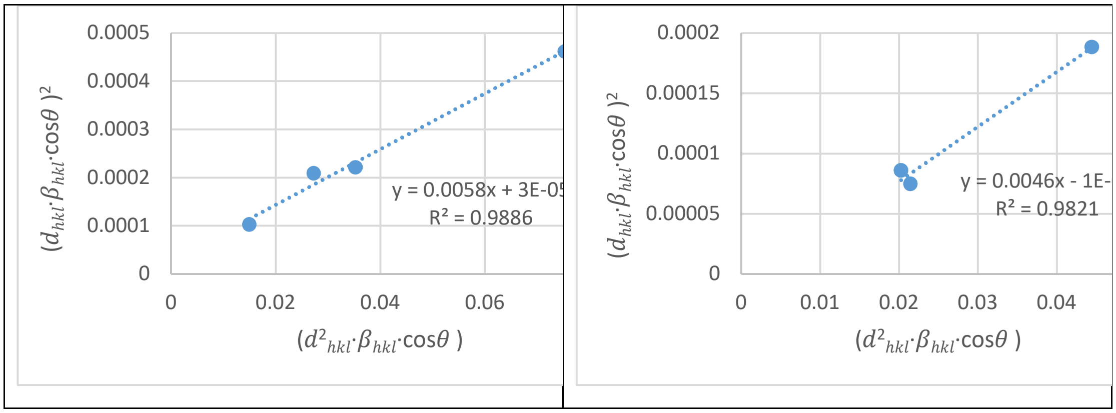

Figure S6. Size-Strain plot for Cr(0.1%)/TiO_2-P25, anatase phase (left), rutile phase (right).

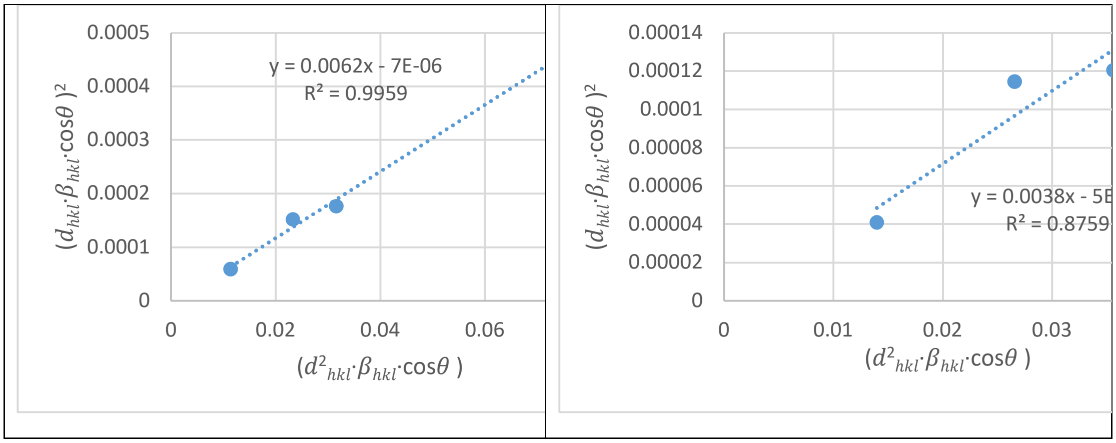

Figure S7. Size-Strain plot for V(0.1%)/TiO_2-P25, anatase phase (left), rutile phase (right).

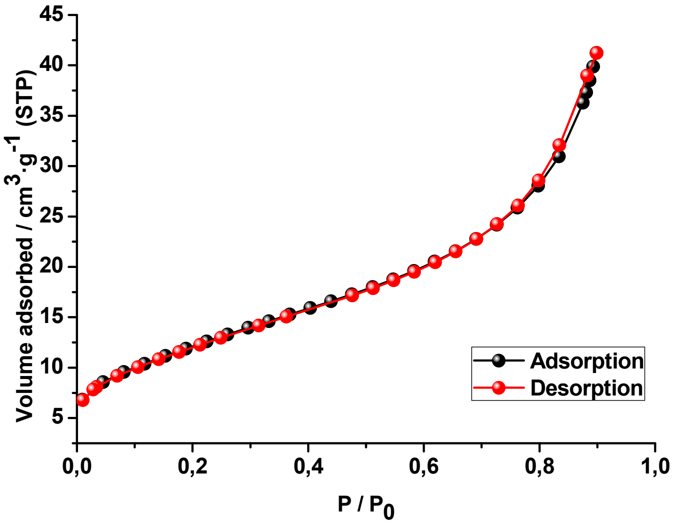

Figure S8. N_2 adsorption-desorption isotherm of V(0.1%)/ TiO_2-P25 photocatalyst.

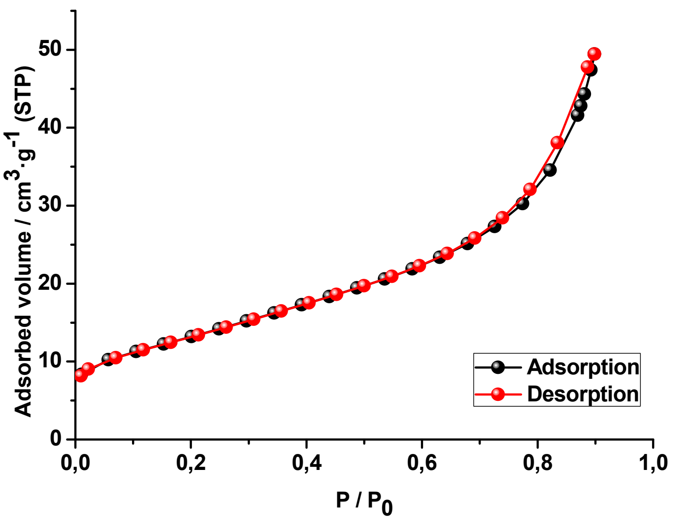

Figure S9. N_2 adsorption-desorption isotherm of Cr(0.1%)/ TiO_2-P25 photocatalyst.

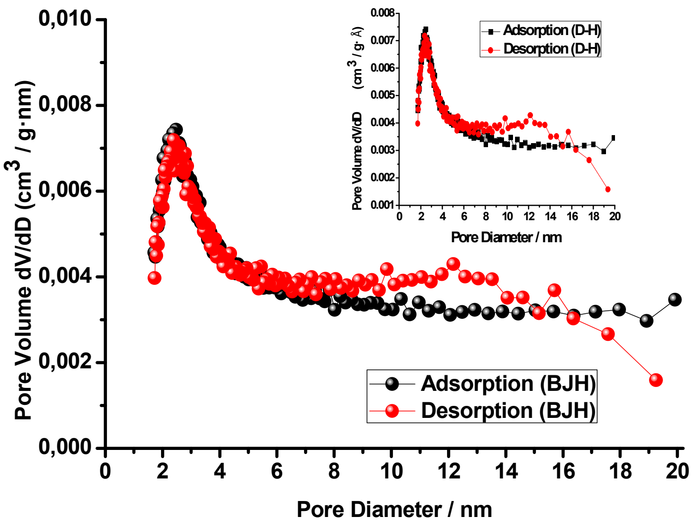

Figure S10. Differential specific pore volume *vs.* pore width distribution for Cu(0.1%)/ TiO_2-P25 photocatalyst using the BJH model. Inset: using the D-H model.

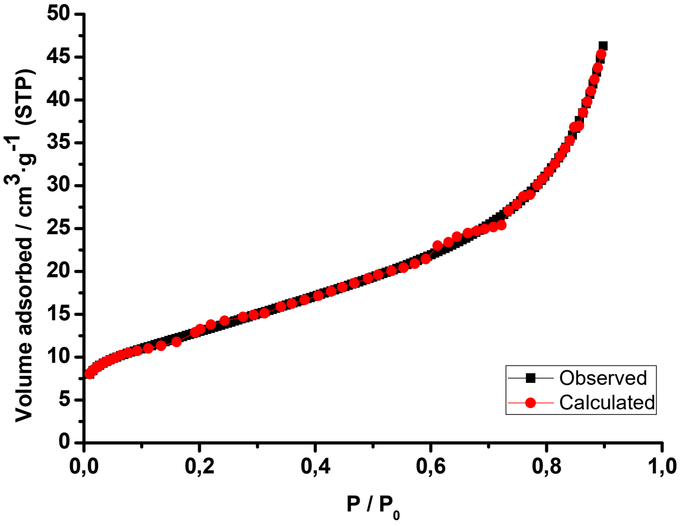

Figure S11. Comparison between observed and calculated isotherm of Cu(0.1%)/ TiO_2-P25 photocatalyst. Calculated isotherm using the 2D-NLDFT model (N_2-Carbon Finite Pores, Aspect Ratio 6, Standard Slit).

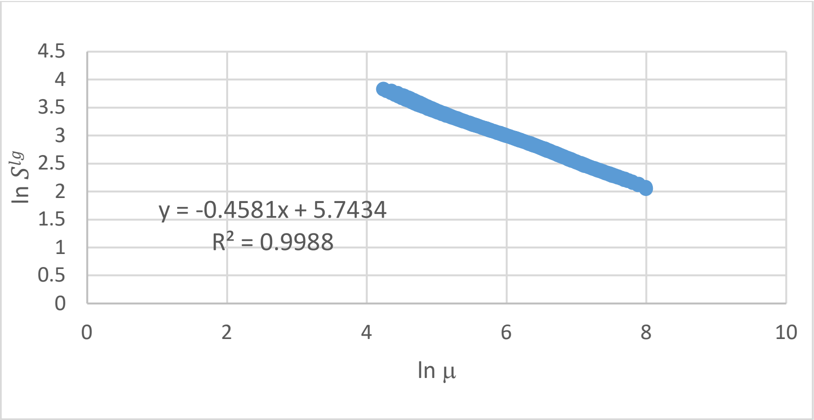

Figure S12. Frenkel-Halsey-Hill fractal analysis of the Cu(0.1%)/TiO_2-P25 isotherm.

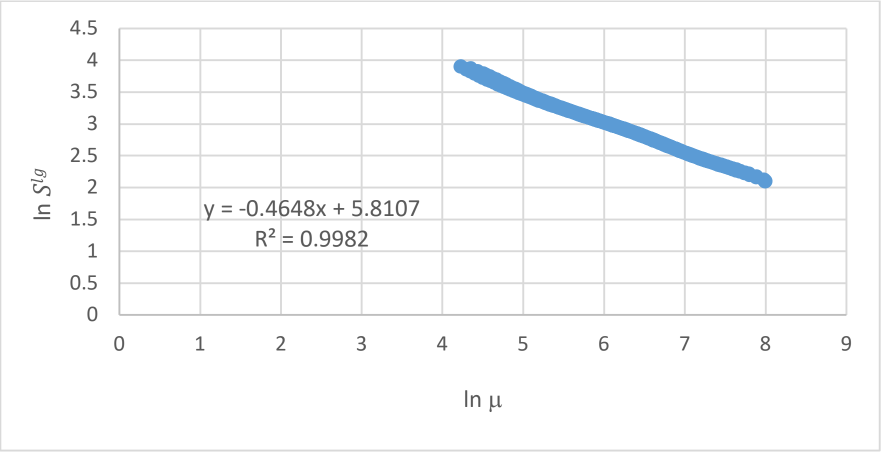

Figure S13. Frenkel-Halsey-Hill fractal analysis of the Cr(0.1%)/TiO_2-P25 isotherm.

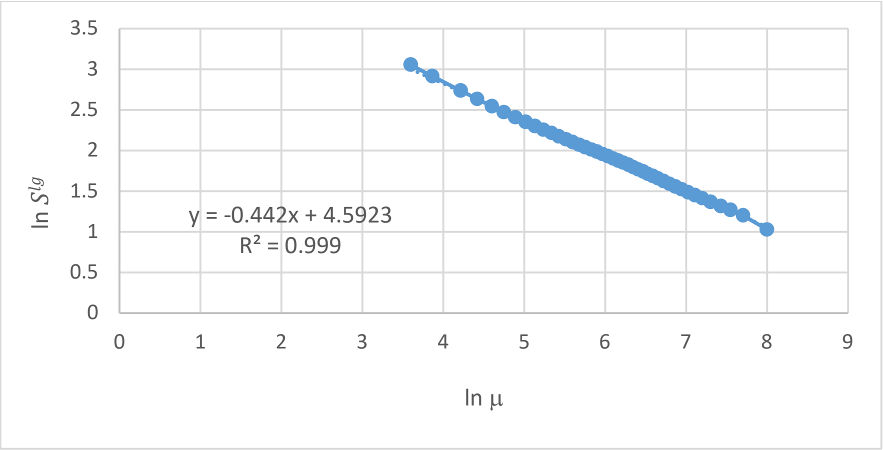

Figure S14. Frenkel-Halsey-Hill fractal analysis of the V(0.1%)/TiO_2-P25 isotherm.

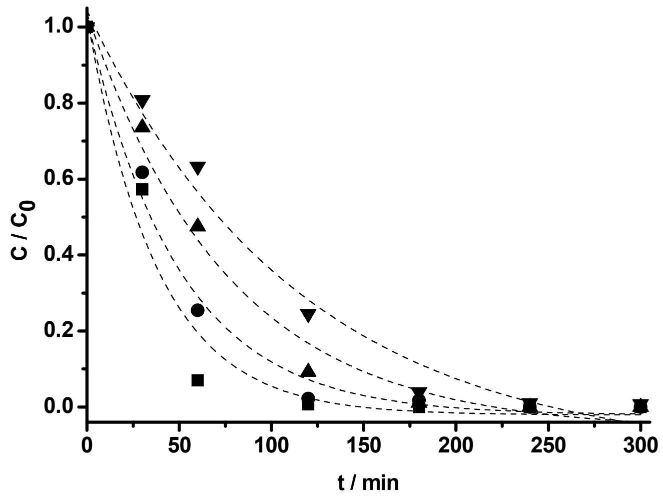

Figure S15. Photodegradation of phenol over Cu(X%)/TiO_2 under NUV-Vis irradiation, UV-Vis spectrophotometric detection. $\lambda_{exc} > 366$ nm. X%: 0.1% (■), 0.3% (●), 0.5% (▲) & 1% (▼). [Phenol]0 = 50 mg·L-1, [Cu(X%)/TiO2]0 = 1.0 g·L-1, natural pH, T = 298.0 K.

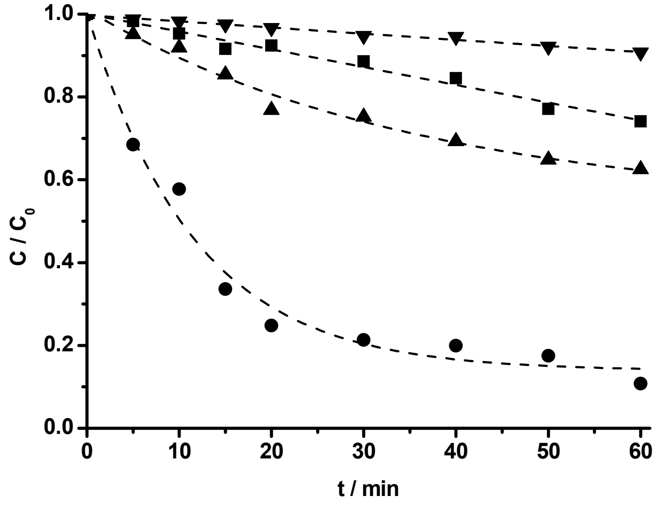

Figure S16. Photodegradation of phenol over Cu(X%)/TiO_2 under UV irradiation, UV-Vis spectrophotometric detection. $\lambda_{exc} = 255$ nm. X%: 0.1% (■), 0.3% (●), 0.5% (▲) & 1% (▼). [Phenol]0 = 50 mg·L-1, [Cu(X%)/TiO2]0 = 1.0 g·L-1, natural pH, T = 298.0 K.

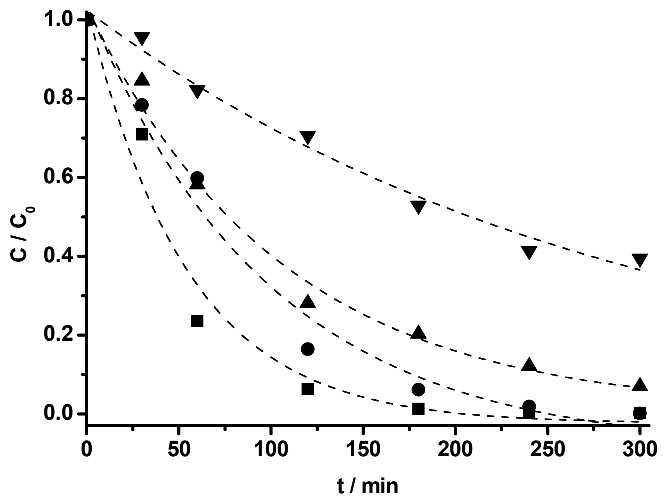

Figure S17. Photodegradation of phenol over V(X%)/TiO_2 under NUV-Vis irradiation, UV-Vis spectrophotometric detection. λ_{exc} > 366 nm. X%: 0.1% (■), 0.3% (●), 0.5% (▲) & 1% (▼). [Phenol]0 = 50 mg·L-1, [V(X%)/TiO2]0 = 1.0 g·L-1, natural pH, T = 298.0 K.

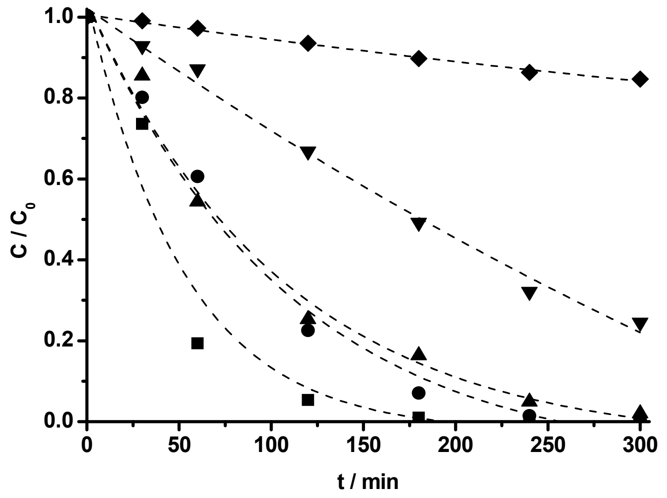

Figure S18. Photodegradation of phenol over V(X%)/TiO_2 under NUV-Vis irradiation. λ_{exc} > 366 nm. HPLC-UV detection (λ = 270 nm). X%: 0% (♦), 0.1% (■), 0.3% (●), 0.5% (▲) & 1% (▼). [Phenol]0 = 50 mg·L-1, [V(X%)/TiO2]0 = 1.0 g·L-1, natural pH, T = 298.0 K.

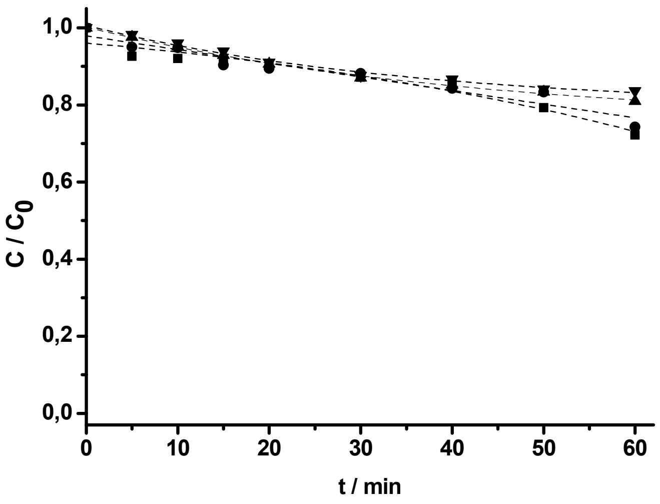

Figure S19. Photodegradation of phenol over V(X%)/TiO_2 under UV irradiation, UV-Vis spectrophotometric detection. λ_{exc} = 255 nm. X%: 0.1% (■), 0.3% (●), 0.5% (▲) & 1% (▼). [Phenol]0 = 50 mg·L-1, [V(X%)/TiO2]0 = 1.0 g·L-1, natural pH, T = 298.0 K.

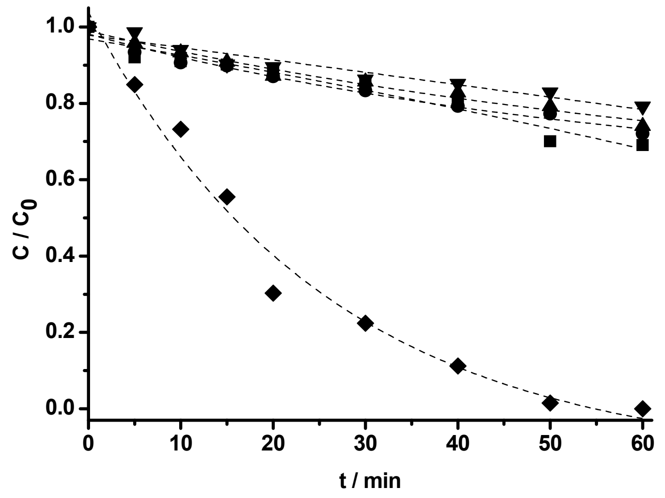

Figure S20. Photodegradation of phenol over V(X%)/TiO_2 under UV irradiation. λ_{exc} = 255 nm. HPLC-UV detection (λ = 270 nm). X%: 0% (♦), 0.1% (■), 0.3% (●), 0.5% (▲) & 1% (▼). [Phenol]0 = 50 mg·L-1, [V(X%)/TiO2]0 = 1.0 g·L-1, natural pH, T = 298.0 K.

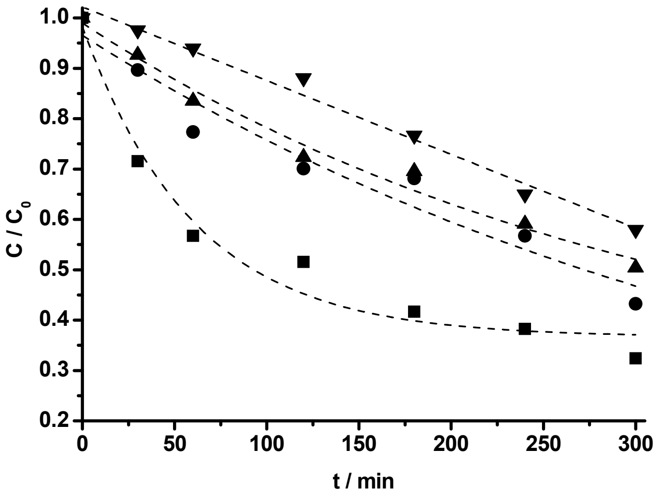

Figure S21. Photodegradation of phenol over Cr(X%)/TiO_2 under NUV-Vis irradiation, UV-Vis spectrophotometric detection. λ_{exc} > 366 nm. X%: 0.1% (■), 0.3% (●), 0.5% (▲) & 1% (▼). [Phenol]0 = 50 mg·L-1, [Cr(X%)/TiO2]0 = 1.0 g·L-1, natural pH, T = 298.0 K.

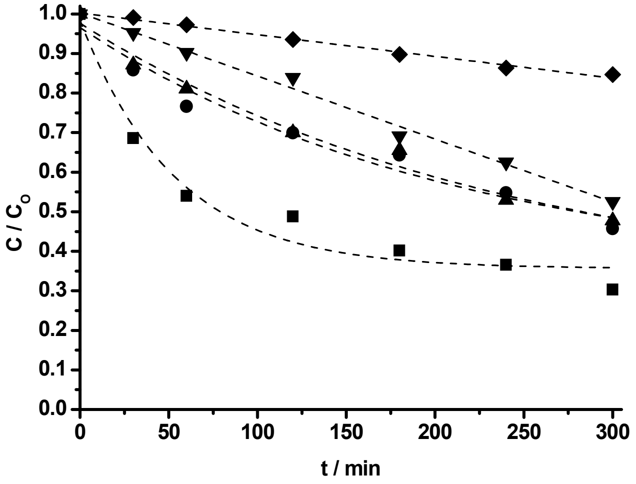

Figure S22. Photodegradation of phenol over Cr(X%)/TiO_2 under NUV-Vis irradiation. λ_{exc} > 366 nm. HPLC-UV detection (λ = 270 nm). X%: 0% (♦), 0.1% (■), 0.3% (●), 0.5% (▲) & 1% (▼). [Phenol]0 = 50 mg·L-1, [Cr(X%)/TiO2]0 = 1.0 g·L-1, natural pH, T = 298.0 K.

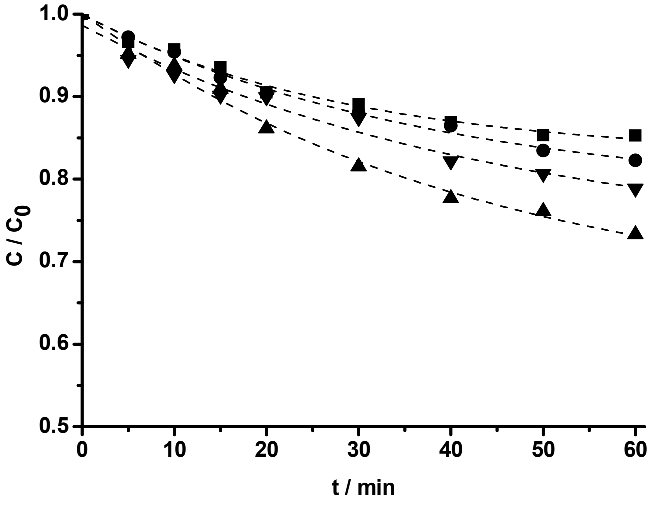

Figure S23. Photodegradation of phenol over Cr(X%)/TiO2 under UV irradiation, UV-Vis spectrophotometric detection. λexc = 255 nm. X%: 0.1% (■), 0.3% (●), 0.5% (▲) & 1% (▼). [Phenol]0 = 50 mg·L-1, [Cr(X%)/TiO2]0 = 1.0 g·L-1, natural pH, T = 298.0 K.

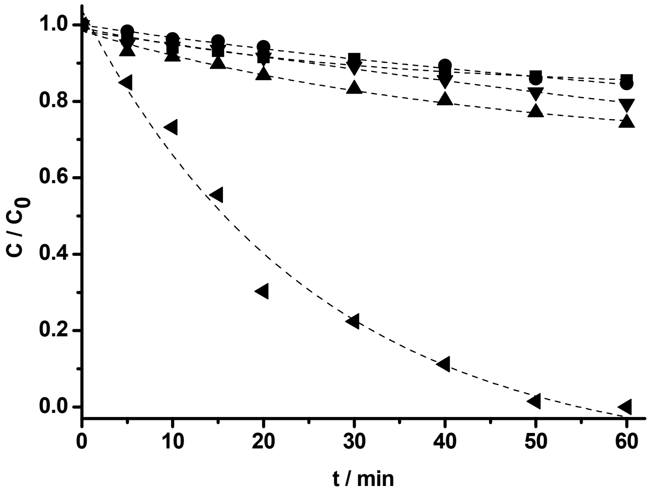

Figure S24. Photodegradation of phenol over Cr(X%)/TiO_2 under UV irradiation. λ_{exc} = 255 nm. HPLC-UV detection (λ = 270 nm). X%: 0% (♦), 0.1% (■), 0.3% (●), 0.5% (▲) & 1% (▼). [Phenol]0 = 50 mg·L-1, [Cr(X%)/TiO2]0 = 1.0 g·L-1, natural pH, T = 298.0 K.
